# Microbial Immobilized Enzyme Biocatalysts for Multipollutant Mitigation: Harnessing Nature’s Toolkit for Environmental Sustainability

**DOI:** 10.3390/ijms25168616

**Published:** 2024-08-07

**Authors:** Mohamed A. A. Abdelhamid, Hazim O. Khalifa, Hyo Jik Yoon, Mi-Ran Ki, Seung Pil Pack

**Affiliations:** 1 Department of Biotechnology and Bioinformatics, Korea University, Sejong-ro 2511, Sejong 30019, Republic of Korea; mohamed42@korea.ac.kr (M.A.A.A.); allheart@korea.ac.kr (M.-R.K.); 2 Department of Botany and Microbiology, Faculty of Science, Minia University, Minia 61519, Egypt; 3 Faculty of Education and Art, Sohar University, Sohar 311, Oman; 4 Department of Veterinary Medicine, College of Agriculture and Veterinary Medicine, United Arab Emirates University, Al Ain P.O. Box 1555, United Arab Emirates; hazimkhalifa@uaeu.ac.ae; 5 Department of Pharmacology, Faculty of Veterinary Medicine, Kafrelsheikh University, Kafr El-Sheikh 33516, Egypt; 6 Institute of Natural Science, Korea University, Sejong-ro 2511, Sejong 30019, Republic of Korea; hyojik88@korea.ac.kr; 7 Institute of Industrial Technology, Korea University, Sejong-ro 2511, Sejong 30019, Republic of Korea

**Keywords:** microbial enzymes, enzyme immobilization, multipollutant contamination, bioremediation, environmental restoration, hydrolases, oxidoreductases

## Abstract

The ever-increasing presence of micropollutants necessitates the development of environmentally friendly bioremediation strategies. Inspired by the remarkable versatility and potent catalytic activities of microbial enzymes, researchers are exploring their application as biocatalysts for innovative environmental cleanup solutions. Microbial enzymes offer remarkable substrate specificity, biodegradability, and the capacity to degrade a wide array of pollutants, positioning them as powerful tools for bioremediation. However, practical applications are often hindered by limitations in enzyme stability and reusability. Enzyme immobilization techniques have emerged as transformative strategies, enhancing enzyme stability and reusability by anchoring them onto inert or activated supports. These improvements lead to more efficient pollutant degradation and cost-effective bioremediation processes. This review delves into the diverse immobilization methods, showcasing their success in degrading various environmental pollutants, including pharmaceuticals, dyes, pesticides, microplastics, and industrial chemicals. By highlighting the transformative potential of microbial immobilized enzyme biocatalysts, this review underscores their significance in achieving a cleaner and more sustainable future through the mitigation of micropollutant contamination. Additionally, future research directions in areas such as enzyme engineering and machine learning hold immense promise for further broadening the capabilities and optimizing the applications of immobilized enzymes in environmental cleanup.

## 1. Introduction

Rapid industrialization and urbanization have exacerbated environmental pollution, making it a critical global challenge [1]. A particular concern lies with micropollutants—persistent contaminants such as pharmaceuticals, dyes, pesticides, and microplastics [2]. These pollutants exhibit remarkable persistence in the environment, posing a significant threat to living organisms and marine life at low concentrations, as evidenced by disruptions in aquatic ecosystems and bioaccumulation within food webs [3,4]. Pharmaceuticals, designed for biological activity, can disrupt microbial communities and promote the emergence of antibiotic resistance [5]. Industrial discharges of dyes not only cause persistent environmental contamination but also obstruct light penetration in water bodies, hindering the growth of photosynthetic organisms and disrupting established food chains [6]. While essential for agriculture, pesticides can leach into soil and water, harming non-target organisms such as beneficial insects and wildlife, and ultimately posing risks to human health through contaminated food sources [7]. Phenolic compounds, known for their widespread presence in industrial effluents and agricultural runoffs, pose significant ecological and health risks by causing oxidative stress in living organisms, disrupting endocrine function, and increasing the incidence of cancers and other chronic diseases in humans [8]. Additionally, plastic and microplastic pollutants are pervasive and persistent, contributing to significant ecological damage and posing a threat to marine life through ingestion and entanglement [9,10]. Therefore, the development of novel and sustainable methods for the removal of these micropollutants is of paramount importance.

A wide range of conventional pollutant removal methods exist, including physical separation techniques (filtration and adsorption), chemical transformation processes (oxidation and precipitation), and biological treatment approaches (activated sludge processes) [11]. However, these established methods are often hindered by several drawbacks, such as high operational costs, insufficient removal of micropollutants, and the potential formation of secondary contaminants with comparable or even greater toxicity, which pose significant challenges [12]. For instance, advanced oxidation processes (AOPs), while effective at degrading complex organic pollutants, can generate toxic byproducts, further complicating environmental remediation efforts. These limitations underscore the critical need for the development of more sustainable and efficient environmental remediation strategies.

Microbial enzymes, a remarkably diverse class of biocatalysts naturally produced by microorganisms, have emerged as a transformative approach to environmental remediation [13]. These enzymes exhibit exceptional chemoselectivity and catalytic efficiency in mediating biochemical reactions critical for pollutant degradation. The unparalleled diversity of microbial life translates to a virtually inexhaustible reservoir of enzymes with the potential to degrade a broad spectrum of environmental contaminants [14]. For instance, laccases and peroxidases demonstrate exceptional efficacy in the breakdown of complex organic molecules such as dyes and pharmaceuticals [15,16,17,18]. Similarly, hydrolases possess the remarkable ability to efficiently degrade a wide range of pesticides [19]. These capabilities are exemplified by the documented degradation of azo dyes, including methyl orange and Reactive Black 5 commonly found in textile industry effluents, as well as widely used agricultural pesticides such as atrazine and chlorpyrifos [19,20]. By harnessing the remarkable catalytic power of these enzymes, we can develop targeted and efficacious strategies for mitigating environmental pollution and promoting long-term environmental sustainability [21]. However, free enzymes, functioning as homogenous biocatalysts, often suffer from inherent limitations that hinder their widespread application in environmental remediation efforts. These limitations include inherent instability, susceptibility to fluctuations in environmental conditions, and challenges associated with their recovery and reuse.

Enzyme immobilization emerges as a compelling solution to address these shortcomings [22]. This technique involves the controlled attachment of enzymes to solid supports, significantly enhancing enzyme stability, activity, and operational longevity [23]. Immobilization provides a protective microenvironment for the enzyme, minimizing degradation and promoting its resilience in fluctuating environmental conditions. Additionally, immobilized enzymes offer the advantage of reusability, enabling cost-effective and sustainable process operations compared to their free enzyme counterparts [24]. A diverse array of immobilization techniques exists, including adsorption, covalent binding, cross-linking, and encapsulation, each offering distinct advantages and limitations that can be optimized for specific environmental applications [25]. Notably, affinity-based immobilization can offer a unique advantage by promoting optimal enzyme orientation on the support material [26,27]. This optimized orientation can significantly enhance the catalytic activity of the enzyme by ensuring proper substrate interaction with the active site [28]. The selection of the most suitable immobilization technique hinges on various factors, including the specific enzyme being employed, the target pollutant, and the desired operational conditions.

**Figure 1:**
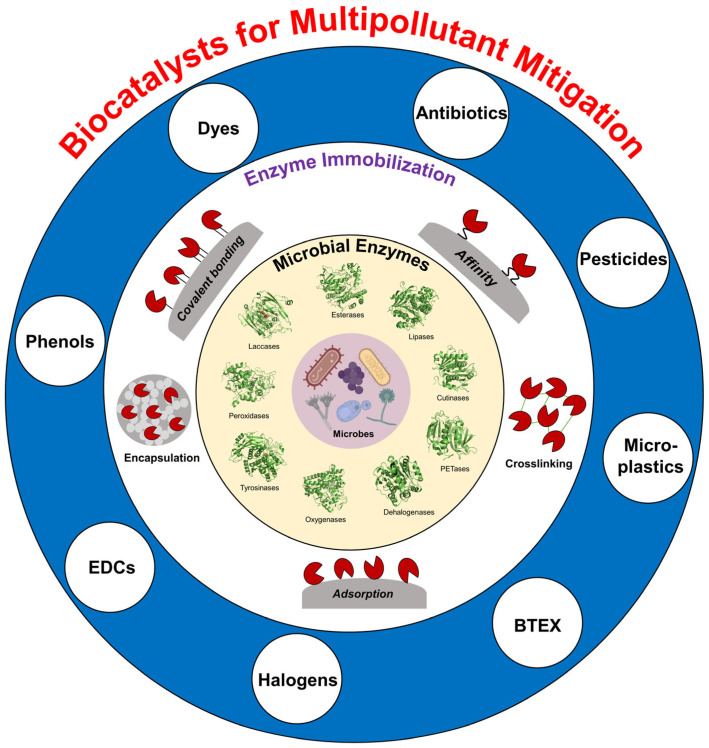
Schematic of immobilized biocatalysts derived from specific microbes for targeted multipollutant degradation.

This review article explores the potential of microbial enzymes as powerful tools for environmental pollution mitigation (Figure 1). We examine the inherent advantages these enzymes possess for degrading micropollutants. Subsequently, the discussion analyzes various immobilization approaches that can significantly enhance enzyme performance. Specific applications of immobilized microbial enzymes for mitigating pharmaceutical pollutants, dyes, and other micropollutants will be highlighted. Through this comprehensive analysis, the review aims to provide a deeper understanding of how enzymes can be harnessed to achieve environmental sustainability.

## 2. Microbial Enzymes for Multipollutant Mitigation

Microbial enzymes offer a diverse and powerful toolkit for mitigating various micropollutants. By harnessing the remarkable biocatalytic capabilities of oxidoreductases (including laccases, peroxidases, tyrosinases, and oxygenases) and hydrolases (encompassing esterases, lipases, cutinases, PETases, and dehalogenases), we possess the potential to achieve the degradation of a broad spectrum of environmental contaminants [29]. These pollutants encompass pharmaceuticals, dyes, pesticides, and a multitude of other complex organic compounds.

### 2.1. Oxidoreductases: Powerful Tools for Pollutant Oxidation

**Figure 2:**
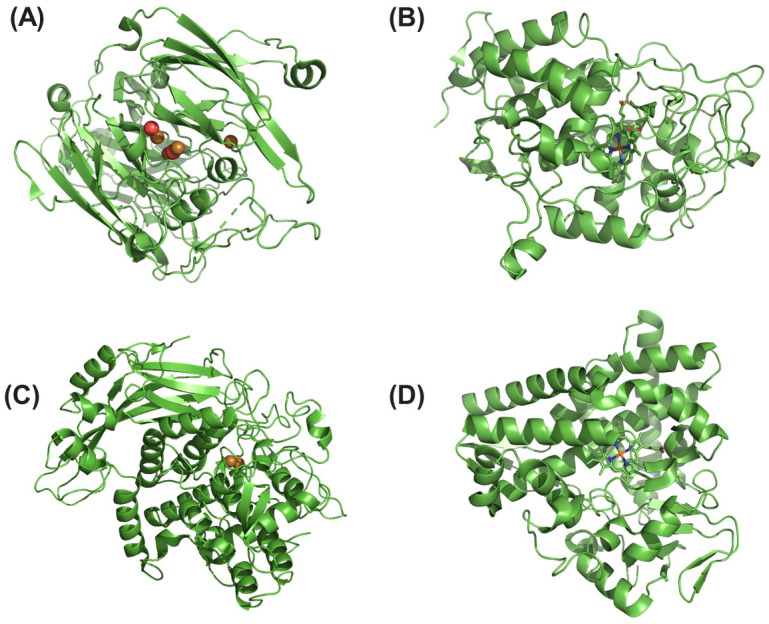
Three-dimensional structures of microbial oxidoreductase enzymes. The selected microbial oxidoreductases are (**A**) *Bacillus subtilis* CotA laccase (PDB ID: 1GSK); (**B**) *Phanerochaete chrysosporium* lignin peroxidase (PDB ID: 1B85); (**C**) one subunit of *Agaricus bisporus* tyrosinase (PDB ID: 5M6B); and (**D**) cytochrome P450 monooxygenase from *Streptomyces scabiei* (PDB ID: 8Q5J).

Microbial oxidoreductases, a diverse group of enzymes known for catalyzing oxidation–reduction reactions, exhibit remarkable efficacy in treating various chemical wastes. These enzymes hold significant promise for environmental remediation, particularly for the degradation of organic micropollutants such as phenols, pharmaceuticals, and hormones [30]. Among these, well-researched oxidoreductases such as laccases, peroxidases, tyrosinases, and oxygenases have garnered significant attention due to their potent biocatalytic capabilities (Figure 2).

#### 2.1.1. Laccases

Laccases (EC 1.10.3.2) constitute a ubiquitous class of multi-copper oxidoreductases found across diverse organisms, including fungi, bacteria, and even some plants [31]. These enzymes have emerged as frontrunners in environmental remediation due to their remarkable ability to oxidize a broad spectrum of aromatic and non-aromatic compounds [32,33]. Their exceptional catalytic prowess is intricately linked to their unique copper architecture.

Laccases harbor three distinct copper centers categorized as type 1 (T1), type 2 (T2), and type 3 (T3) [34]. The T1 copper center, a mononuclear site, serves as the primary electron acceptor from the targeted substrate undergoing oxidation [35]. Conversely, the T2 and T3 copper centers form a trinuclear cluster, playing a crucial role in binding and reducing molecular oxygen to water. The redox potential disparity between the T1 copper and the substrate governs the efficiency of laccase-mediated oxidation. Compounds exhibiting a lower ionization potential (facilitating electron donation) are preferentially oxidized by laccases [35]. This inherent property dictates the range of substrates that a specific laccase can effectively target. However, it is crucial to recognize that for certain recalcitrant pollutants with high redox potentials, laccases may require the assistance of mediators [36]. These mediators, often small molecules with lower redox potentials than the target pollutant, act as electron shuttles. They facilitate the transfer of electrons from the laccase to the pollutant, ultimately enhancing its breakdown process. Common examples include synthetic mediators such as 2,2′-azino-bis(3-ethylbenzothiazoline-6-sulfonic acid) (ABTS) and 1-hydroxybenzotriazole (HBT), both known for their broad effectiveness [36]. Additionally, naturally occurring mediators like syringaldazine, derived from lignin degradation, can be particularly effective against specific pollutants [37]. Selecting the optimal mediator hinges on a complex interplay between the specific laccase employed, the targeted micropollutant’s characteristics, and environmental factors such as pH and temperature.

Microbial laccases, produced by both fungi and bacteria, are emerging as powerful biocatalysts for environmental remediation [33,38]. Fungal laccases, particularly those from *Trametes* and *Pleurotus* genera, have garnered significant interest due to their remarkable ability to degrade a wide range of pollutants, including complex dyes and pharmaceuticals [39,40,41]. Research has demonstrably established the effectiveness of laccase isolated from *Trametes versicolor* in breaking down Remazol Brilliant Blue R dye, carbamazepine, and acetaminophen [42,43]. In contrast, laccase from *Pleurotus ostreatus* exhibits activity against Congo Red dye and chlorophenols, highlighting their broad substrate specificity [44,45]. This inherent versatility positions fungal laccases as promising tools for mitigating diverse environmental pollutants.

While fungal laccases have received much attention, bacteria offer another exciting avenue for laccase-based bioremediation. Bacterial laccase activity was first detected in *Azospirillum lipoferum* in 1993 [46], and laccases have since been discovered in various bacterial genera, such as *Bacillus* [47], *Streptomyces* [48], *Klebsiella* [49], *Pseudomonas* [50], *Yersinia* [51], *Proteobacterium* [52], and *Marinomonas* [53]. These enzymes, typically ranging from 50 to 70 kDa in size and existing as either monomeric extracellular or intracellular proteins, play various roles in bacterial processes, including pigmentation, toxin oxidation, morphogenesis, and protection against UV light and oxidizing agents [54,55]. Notably, the CotA protein of *B. subtilis* is a unique copper-dependent laccase present in the spore coat, highlighting the diverse roles these enzymes can play within bacteria [56].

#### 2.1.2. Peroxidases

Peroxidases (EC 1.11.1.x) are a diverse group of enzymes found throughout nature, playing vital roles in various organisms, including plants, animals, fungi, and bacteria [57]. These versatile enzymes are garnering significant attention for their potential applications in environmental remediation, particularly due to their robust oxidative capabilities that enable them to degrade a broad range of recalcitrant pollutants [58,59]. Structurally, peroxidases rely on a heme prosthetic group as the cornerstone of their catalytic activity. This heme group, consisting of an iron ion complexed within a porphyrin ring, facilitates the electron transfer reactions essential for substrate oxidation [60]. Variations in the surrounding amino acid residues influence the diverse substrate specificities and catalytic efficiencies observed across different peroxidases [61].

A remarkable diversity of microbial peroxidases exists in nature, each with specific functionalities. Found primarily in white-rot fungi such as *Phanerochaete chrysosporium*, lignin peroxidases (LiPs) excel at breaking down complex pollutants, including lignin [62]. Meanwhile, manganese peroxidases (MnPs), produced by fungi such as *T. versicolor*, oxidize Mn^2+^ to Mn^3+^, which then tackles various organic substrates [63]. *Pleurotus ostreatus* stands out with its versatile peroxidases (VPs), which combine the capabilities of both LiPs and MnPs, enabling them to address a wider range of pollutants [64].

Bacteria also contribute significantly to the peroxidase repertoire. *Bacillus stearothermophilus* produces catalase-peroxidases (KatGs), enzymes with dual functionality in hydrogen peroxide degradation and stress response [65]. *Pseudomonas putida* wields peroxidases specifically designed for the degradation of aromatic compounds, a crucial function in bioremediation efforts aimed at decontaminating polluted environments [66]. *Streptomyces* spp., renowned for their antibiotic production, also produce peroxidases that play a crucial role in organic compound breakdown, highlighting the multifaceted nature of these enzymes [67]. Furthermore, yeasts such as *Saccharomyces cerevisiae* and *Candida* spp., possess peroxidases that enhance their environmental resilience [68,69]. These enzymes highlight the versatility of peroxidases across different organisms and their potential for future applications.

#### 2.1.3. Tyrosinases

Tyrosinases (EC 1.14.18.1) are copper-containing enzymes found across a diverse range of microorganisms, including bacteria and fungi. These enzymes play a crucial role in the biosynthesis of melanin and other polyphenolic compounds through the oxidation of phenols [70]. Tyrosinases have garnered significant interest due to their potential applications in fields such as bioremediation, pharmaceuticals, and the food industry [71].

Tyrosinases play a crucial role in micropollutant mitigation by catalyzing the oxidation of phenolic contaminants found in industrial wastewater [72]. These enzymes transform monophenols into o-diphenols and subsequently into o-quinones, utilizing a binuclear copper center for their catalytic activity [72]. The resulting o-quinones are more reactive and can further polymerize into less harmful compounds, facilitating their removal from the environment. This enzymatic process not only reduces the toxicity of phenolic pollutants but also aids in their complete degradation, contributing significantly to the detoxification and remediation of contaminated water sources [73].

Microbial tyrosinases are highly valued for their stability, ease of production, and potential applications in bioremediation. For instance, *Agaricus bisporus* (common mushroom) produces tyrosinases that are extensively studied for their effectiveness in breaking down phenolic compounds in polluted environments, making them highly useful for bioremediation [74,75,76]. Similarly, *Streptomyces glaucescens* produces a well-characterized tyrosinase known for its high activity and robustness, which is particularly valuable for treating phenolic wastewater and degrading harmful pollutants [70]. Additionally, *Bacillus megaterium* produces tyrosinases that are employed in the degradation of phenolic pollutants, contributing significantly to the detoxification of contaminated sites by breaking down complex phenolic structures [77]. These microorganisms demonstrate the substantial potential of microbial tyrosinases in environmental cleanup and bioremediation efforts.

#### 2.1.4. Oxygenases

Oxygenases, a diverse group of enzymes, play a vital role in nature by incorporating oxygen atoms from molecular oxygen (O_2_) into organic substrates [78]. These enzymes fall into two main categories: monooxygenases (EC 1.14.x.x) and dioxygenases (EC 1.13.x.x). While monooxygenases incorporate one oxygen atom and reduce the other to water, dioxygenases incorporate both atoms directly into the substrate [79]. Oxygenases, essential enzymes found ubiquitously in nature, play a critical role in diverse processes, including xenobiotic metabolism, natural product biosynthesis, and even environmental pollutant degradation [80,81]. Their remarkable ability to catalyze diverse oxidative reactions positions them as highly valuable tools for bioremediation strategies targeting organic micropollutants. This versatility offers a promising approach to the sustainable decontamination of polluted environments.

Oxygenases, encompassing monooxygenases dioxygenases, utilize distinct yet complementary mechanisms to manipulate oxygen for organic substrate degradation. Monooxygenases initiate the process with substrate binding and activation at the enzyme’s active site. A cofactor like NADH, NADPH, or FAD then facilitates the reduction in molecular oxygen, leading to the incorporation of one oxygen atom into the substrate, forming a hydroxylated product [82,83]. The remaining oxygen atom is reduced to water, completing the catalytic cycle and releasing the modified substrate. Dioxygenases, on the other hand, often rely on metal ions such as Fe^2+^ or Mn^2+^ to activate molecular oxygen upon substrate binding. This activated oxygen then undergoes complete incorporation into the substrate, resulting in either a dihydroxylated product or a cleaved ring structure [84]. Following product release, both monooxygenases and dioxygenases are regenerated for subsequent rounds of catalysis. These distinct but versatile mechanisms equip oxygenases with the remarkable ability to perform a broad spectrum of oxidative transformations, making them highly effective tools for the bioremediation of complex organic micropollutants [85].

The remarkable diversity of microbial communities underpins the production of oxygenases. Bacteria, fungi, and yeasts collectively possess a diverse enzymatic capacity that facilitates the biodegradation of a broad spectrum of environmental micropollutants. Bacterial species such as *P. putida*, *Rhodococcus jostii*, and *Mycobacterium* sp., frequently isolated from contaminated environments, are renowned oxygenase producers [86,87,88]. These bacteria demonstrate remarkable proficiency in the degradation of aromatic hydrocarbons and other organic pollutants present in soil and water environments. Similarly, specific fungal species, such as *P. chrysosporium* and *P. ostreatus*, exhibit high efficacy in lignin and complex organic substance degradation [89,90]. Additionally, yeasts, such as *Candida parapsilosis* and *Candida albicans*, contribute to the bioremediation toolbox [91,92]. These yeasts produce oxygenases with remarkable stability and broad substrate specificity [91,92]. By harnessing the oxygenase-producing potential of these diverse microbial sources, we can develop efficient strategies for bioremediating a wide range of organic micropollutants [93].

### 2.2. Hydrolases

**Figure 3:**
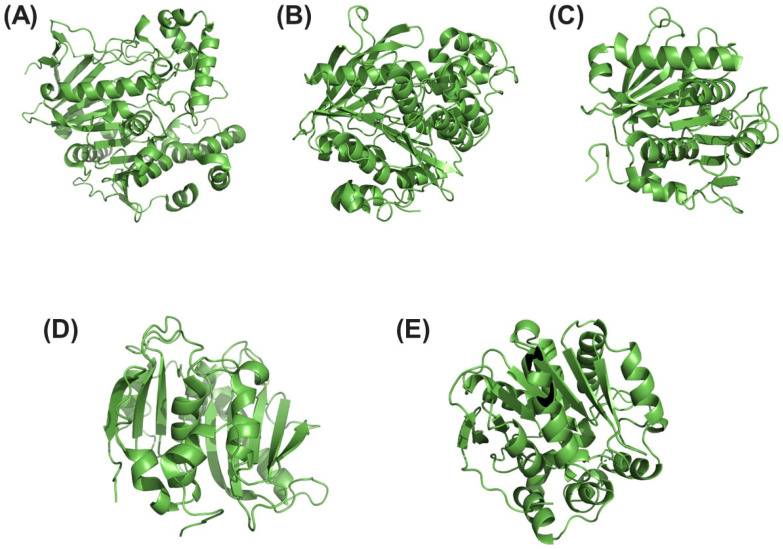
Three-dimensional structures of microbial hydrolase enzymes. The selected microbial hydrolases are (**A**) *Bacillus subtilis* esterase (PDB ID: 1QE3); (**B**) *Candida antarctica* lipase A (PDB ID: 2VEO); (**C**) *Thermobifida fusca* cutinase (PDB ID: 5ZOA); (**D**) *Ideonella sakaiensis* PETase (PDB ID: 5XJH); and (**E**) haloalkane dehalogenase from a *Rhodococcus* species (PDB ID: 1BN6).

Hydrolases, a versatile class of enzymes, offer a promising avenue for mitigating micropollutants in the environment [94]. They function as biological catalysts, facilitating the hydrolysis of complex contaminants such as pesticides, pharmaceuticals, and FOG (fats, oils, and grease) [95]. This process utilizes water molecules to cleave specific bonds within the pollutant structure, effectively breaking them down into simpler, less harmful components. This targeted approach provides a sustainable and efficient strategy for environmental remediation. Notably, different subclasses within the hydrolase family possess distinct substrate specificities. Enzymes such as esterases, lipases, cutinases, PETases, and dehalogenases exhibit high substrate specificity (Figure 3). This characteristic allows them to target and degrade specific types of pollutants, thereby minimizing unintended environmental consequences for non-target molecules [96,97]. This bioremediation strategy leveraging the power of hydrolases holds significant potential for achieving a cleaner and healthier environment.

#### 2.2.1. Esterases

Esterases (EC 3.1.1.x) are a class of hydrolase enzymes that catalyze the hydrolysis of ester bonds, converting esters into their corresponding acids and alcohols [98]. These enzymes are ubiquitous in nature and found in a wide range of organisms, including plants, animals, and microorganisms [98]. Esterases play crucial roles in various biological processes, such as lipid metabolism, detoxification of xenobiotics, and the degradation of complex organic molecules. Due to their broad substrate specificity and ability to function under mild conditions, esterases have garnered significant attention for their potential applications in environmental remediation, particularly in the breakdown of micropollutants [96].

Esterases operate through a well-coordinated catalytic mechanism reliant on a conserved triad of amino acid residues: serine, histidine, and aspartate. This catalytic triad facilitates a nucleophilic attack on the carbonyl carbon atom of the ester bond [99]. The esterase-mediated hydrolysis proceeds in a stepwise manner. Initially, the serine hydroxyl group acts as a nucleophile, attacking the carbonyl carbon and forming a transient, tetrahedral intermediate [100]. Subsequently, the intermediate collapses, releasing the alcohol moiety from the ester substrate and generating an acyl-enzyme intermediate, where the remaining fragment of the ester is covalently bound to the enzyme. Finally, a water molecule participates in the reaction, hydrolyzing the acyl-enzyme intermediate [101]. This hydrolysis step releases the carboxylic acid component of the original ester molecule and regenerates the free enzyme, primed for another catalytic cycle. This efficient catalytic machinery enables esterases to effectively degrade a diverse range of ester-containing micropollutants [94,101].

A diverse array of microorganisms, including bacteria, fungi, and yeasts, serve as prolific sources of esterases [102]. This biocatalytic potential makes them valuable tools for industrial and environmental applications [103]. Several bacterial species, including *Pseudomonas aeruginosa*, *B. subtilis*, and *Escherichia coli*, are well-established esterase producers [104,105,106]. Fungi such as *Aspergillus niger* and *Penicillium chrysogenum* demonstrate exceptional capabilities in producing robust esterases capable of degrading complex organic substrates [107,108]. The ability of these fungi to secrete large quantities of extracellular enzymes renders them particularly valuable for bioremediation efforts [109]. Yeasts, exemplified by *Candida rugosa* and *Pseudozyma antarctica*, further contribute to the diversity of esterase-producing microorganisms [110,111]. These microorganisms can be strategically employed in bioreactors or bioaugmentation strategies to significantly enhance the degradation of ester-containing pollutants in contaminated environments [112].

#### 2.2.2. Lipases

Lipases (EC 3.1.1.3), another versatile class of hydrolase enzymes, are nature’s experts in fat breakdown [113]. Lipases, a ubiquitous class of enzymes, are widely distributed across diverse organisms, including animals, plants, and microorganisms [114]. Their primary function is the hydrolysis of triglycerides, the major storage form of fats and oils. This hydrolysis reaction yields glycerol and free fatty acids, effectively dismantling these lipid molecules. Beyond their role in fat digestion and metabolism within organisms, lipases offer significant potential for various industrial applications [113]. Their remarkable versatility, allowing them to act on a wide range of lipid substrates, and their ability to function under diverse environmental conditions make them ideal candidates for biodiesel production, food processing, and even bioremediation of environments contaminated with lipid-rich pollutants [95,115,116].

Lipases (triacylglycerol hydrolases) exhibit a unique catalytic mechanism tailored for the hydrolysis of ester bonds within triglycerides, differentiating them from general esterases [117]. This two-step process, termed acylation and deacylation, is mediated by a conserved catalytic triad of serine, histidine, and aspartate residues, similar to their esterase counterparts [118]. A critical distinction lies in the initial activation step. Unlike esterases, lipases require an interfacial activation triggered by the presence of a water–lipid interface, a prevalent feature in contaminated environments (e.g., oil–water interface). This interfacial activation induces a conformational change within the enzyme structure, priming it for subsequent catalysis [119]. Following activation, the acylation step commences. The serine hydroxyl group within the active site functions as a nucleophile, launching a nucleophilic attack on the carbonyl carbon of the triglyceride’s ester bond. This attack results in the formation of a transient tetrahedral intermediate. The intermediate subsequently collapses, releasing one fatty acid and generating an acyl-enzyme intermediate [100]. In the deacylation step, a water molecule participates in the hydrolysis of the acyl-enzyme intermediate, liberating the second fatty acid. This step regenerates the free lipase, readying it for another catalytic cycle. This efficient two-step mechanism, coupled with the interfacial activation requirement, equips lipases to effectively degrade triglycerides and other lipid substrates. This unique functionality renders lipases invaluable biocatalysts for various bioremediation applications [95].

Microbial lipases, nature’s frontline biocatalysts for decontaminating lipid-rich pollutants, originate from a remarkably diverse array of microorganisms [120]. Bacterial species such as *P. aeruginosa*, frequently isolated from contaminated environments, along with *B. subtilis* and *Burkholderia cepacia*, are well-established lipase producers [121,122]. Their ubiquitous presence in polluted ecosystems suggests an intrinsic role for their lipases in natural detoxification processes. The fungal kingdom harbors equally potent lipase producers, excelling in extracellular enzyme production. *Aspergillus niger*, *Rhizopus oryzae*, and *Mucor miehei* are prominent examples, offering a distinct advantage for large-scale production due to their exceptional secretory abilities [123]. These commercially available fungal lipases find utility in various sectors like detergent formulation, food processing, bioremediation, and biodiesel production [123]. Furthermore, yeasts like *C. antarctica* and *Yarrowia lipolytica* are renowned for their remarkable stability and broad substrate specificity, allowing them to effectively target a wider range of pollutants in contaminated environments [124,125]. By harnessing this remarkable diversity of microbial lipases, researchers can develop powerful strategies to enhance the degradation of lipid-rich pollutants. These enzymes, derived from a wide range of microorganisms, offer a sustainable and environmentally friendly solution for ecosystem restoration. Their inherent biodegradability and minimal impact on non-target organisms solidify their position as invaluable tools for bioremediation efforts.

#### 2.2.3. Cutinases

Cutinases (EC 3.1.1.74) belong to the serine esterase family and are produced by fungi and bacteria [126]. These enzymes play a vital role in the natural decomposition process by hydrolyzing cutin, a complex polyester component of the plant cuticle. However, their remarkable broad substrate specificity extends beyond their natural substrate, effectively degrading a diverse range of synthetic polyesters and triglycerides [127,128]. Notably, cutinases stand out as nature’s frontline biocatalysts, capable of effectively breaking down a wide range of synthetic polyesters and toxic xenobiotics such as polyethylene terephthalate (PET), polylactic acid (PLA), polycaprolactone (PCL), polyhydroxybutyrate succinate (PBS), phthalate esters, and malathion esters [129]. Their ability to break down complex polymeric substances underscores their value as tools for restoring polluted environments.

Cutinases catalyze the hydrolysis of ester bonds in a two-step process involving a covalent intermediate and hydrolyzing ester bonds [130]. A catalytic serine residue first attacks the ester bond, forming a transient tetrahedral intermediate and an acyl-enzyme complex. Subsequently, a water molecule activated by histidine cleaves this complex, releasing a carboxylic acid and regenerating the free enzyme.

Microbial diversity serves as a rich reservoir for cutinase production, enzymes with remarkable potential for micropollutant degradation. These biocatalysts are primarily produced by fungi and bacteria that naturally inhabit environments rich in plant materials or contaminated with synthetic polyesters. Fungal species such as *Fusarium solani*, *Aspergillus nidulans*, and *Humicola insolens* are well-established cutinase producers [129]. Similarly, bacterial strains, including *Thermobifida fusca*, *Pseudomonas putida*, and *Botrytis cinerea,* demonstrate cutinase production capabilities [129]. The isolation of these microorganisms from environments polluted with synthetic polyesters and other polymers suggests an adaptation to utilize these anthropogenic materials as a carbon source [130]. By leveraging this remarkable enzymatic diversity from both fungal and bacterial sources, researchers can catalyze a new approach to bioremediation, leading to a future free from micropollutant burdens.

#### 2.2.4. PETase and MHETase

PETase (EC 3.1.1.101) is an enzyme analogous to the cutinase family, discovered in the bacterium *Ideonella sakaiensis*, which degrades polyethylene terephthalate (PET) into mono(2-hydroxyethyl) terephthalate (MHET) and terephthalic acid (TPA) [131]. Structurally, PETase features an α/β hydrolase fold with a highly exposed active site, distinct from cutinase, and contains a catalytic triad of Ser160, Asp206, and His237 [132]. PETase can cleave polymeric chains both endo- and exo-fashion, allowing it to degrade PET and polyethylene-2,5-furandicarbonate (PEF) but not aliphatic polyesters [133].

MHETase (EC 3.1.1.102), co-produced by *Ideonella sakaiensis* during PET degradation, hydrolyzes MHET into TPA and ethylene glycol (EG) [131]. The enzyme’s structure includes five disulfide bonds, with a catalytic triad of S225, D492, and H528, and an oxyanion hole formed by G132 and E226. MHETase’s specific affinity for the para-carboxy group of its substrate underscores its precise role in the PET degradation pathway [134]. Although its classification remains ambiguous between feruloyl esterase and tannase, MHETase’s function is crucial in completing the conversion of PET waste into reusable monomers. It works synergistically with PETase to achieve efficient plastic degradation.

#### 2.2.5. Dehalogenases

Dehalogenases (EC 3.8.1.5) represent a diverse class of enzymes renowned for their remarkable ability to catalyze the removal of halogen atoms (chlorine, bromine, fluorine, and iodine) from organic compounds [135]. These enzymes play a critical role in the biodegradation of halogenated organic pollutants, a category encompassing a wide range of industrial chemicals, pesticides, and solvents notorious for their persistence and toxicity in the environment [136,137]. Microbial dehalogenases exhibit remarkable mechanistic diversity, showcasing their remarkable adaptability for halogenated compound degradation. This heterogeneity is strikingly evident within the enzymatic repertoire of a single bacterial species, *Rhizobium* sp. [138]. Notably, dehalogenases DehL, DehD, and DehE exemplify this impressive diversity. Each enzyme demonstrates distinct substrate enantioselectivity (preference for L- or D-halocarboxylic acids) and catalytic efficiency, highlighting the nuanced biochemical adaptations within this microorganism. Although all three enzymes utilize an S_N_2 nucleophilic substitution mechanism to displace the halide ion, their detailed reaction pathways diverge. DehL employs a two-step process, forming an ester intermediate prior to hydrolysis. Conversely, DehD and DehE directly activate a water molecule for halide substitution, bypassing the ester intermediate formation step. This remarkable enzymatic diversity within a single bacterial species underscores the profound adaptability of microbial catalysts and their potential applications in various environmental bioremediation strategies.

Microbial dehalogenases represent a diverse and potent suite of enzymes produced by a broad taxonomic spectrum of bacteria (e.g., *Dehalococcoides mccartyi*, *Desulfitobacterium hafniense*, and *P. aeruginosa*) and fungi (e.g., *P. chrysosporium*, and *T. versicolor*) [136,139,140,141]. These enzymes exemplify the remarkable adaptability of microbial communities in response to environmental perturbations, particularly those involving halogenated compound contamination. Notably, microorganisms isolated from such polluted environments frequently harbor dehalogenases with a versatile repertoire of dehalogenation mechanisms (hydrolytic, reductive, and oxidative) for the bioremediation of these recalcitrant pollutants. By leveraging this inherent biocatalytic potential of dehalogenase-producing microbes, researchers can develop efficacious strategies for the detoxification and restoration of polluted ecosystems.

## 3. Microbial Enzyme Immobilization

### 3.1. Immobilization Approaches for Enhancing Enzyme Performance

**Figure 4:**
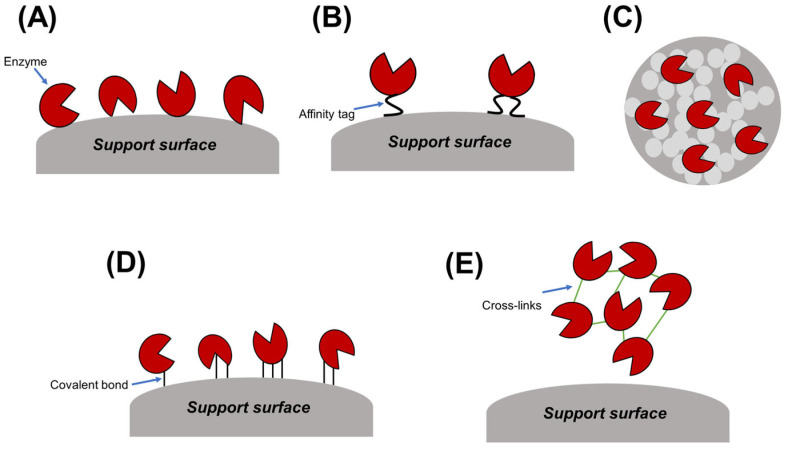
Immobilization techniques for microbial enzymes. The figure illustrates primary immobilization methods: (**A**) adsorption, (**B**) affinity-based approach, (**C**) encapsulation, (**D**) covalent bonding, and (**E**) cross-linking. These techniques enhance enzyme stability and reusability for efficient pollutant degradation.

Microbial enzymes represent a promising avenue for bioremediation due to their inherent ability to catalyze the degradation of diverse environmental pollutants. However, their practical applicability can be hindered by limitations in operational stability and reusability. For instance, the lipase enzyme derived from *Burkholderia glumae* exhibits limited stability, often deactivating under varying temperatures, pH, and solvent conditions [142]. Similarly, the laccase enzyme derived from *Aspergillus oryzae* is susceptible to deactivation under extreme environmental conditions [143]. Immobilization techniques emerge as a transformative strategy to address these challenges (Figure 4). By anchoring enzymes onto activated or inert supports, immobilization enhances their stability, safeguarding them from harsh environmental conditions and thereby extending their functional half-life. This not only translates to improved efficiency in pollutant degradation but also facilitates their repeated use, leading to significant cost reductions and minimization of enzyme waste. Notably, various immobilization approaches, including adsorption, encapsulation, covalent attachment, cross-linking, and affinity-based methods, offer researchers the ability to tailor the properties of immobilized enzymes for specific environmental applications. This targeted approach allows for the development of effective bioremediation strategies that can address a wide range of environmental challenges.

#### 3.1.1. Adsorption Immobilization

Adsorption presents a well-established and versatile technique for optimizing enzyme utilization [144]. This method employs non-covalent interactions, such as ionic bonds, hydrophobic interactions, hydrogen bonds, and van der Waals forces, to physically anchor enzymes onto a diverse array of support materials [145]. Enzyme adsorption techniques exploit a diverse array of carrier materials, categorized broadly into organic and inorganic origins. Common inorganic carriers include silica, titania, and hydroxyapatite, recognized for their exceptional robustness and stability in supporting immobilized enzymes [146]. Organic carriers, conversely, encompass a wider spectrum, including natural biopolymers such as chitin, chitosan, cellulose, and alginate, alongside synthetic counterparts such as polystyrene and polyacrylamide [144]. Notably, advanced nanomaterials like graphene oxide and Ti_3_C_2_ MXene nanosheets represent a burgeoning frontier in enzyme immobilization strategies [147,148]. The primary advantage of these matrices lies in their inherent tailorability. They can be readily modified at the chemical level to precisely match the optimal conditions required for a specific enzyme and its intended application, ultimately leading to enhanced enzyme performance and stability [144]. This allows for the efficient attachment of enzyme molecules to the support surface through physical attractions.

Surface immobilization offers several advantages that contribute to its widespread adoption. Unlike some techniques, it utilizes mild conditions that minimize the potential denaturation of the enzyme, thereby preserving its original structure and activity. Additionally, surface-immobilized enzymes possess the significant benefit of being easily recovered and reused after a reaction, leading to a substantial reduction in operational costs associated with enzyme replacement. The inherent simplicity of the attachment process also facilitates easy scale-up for industrial applications, making it a practical choice for large-scale biocatalytic processes. However, enzyme leaching represents a significant challenge in surface immobilization. Over time, the non-covalent interactions holding the enzyme to the support material may weaken, leading to the detachment and loss of the enzyme into the surrounding solution. This results in a decrease in immobilized enzyme activity and necessitates replenishing the enzymes or replacing the support material. For instance, a study investigating the immobilization of α-amylase from *Aspergillus fumigatus* on zeolite support observed a dramatic decrease in its catalytic activity over reusability cycles, retaining only 13% of the initial activity after five reuse cycles [149]. This example underscores the importance of meticulous selection of carrier materials and optimization of immobilization protocols to minimize enzyme leaching and ensure long-term operational stability.

Despite this potential limitation, surface immobilization remains a valuable approach, particularly for applications where enzyme reusability and facile separation from the reaction mixture are paramount. For example, graphene oxide, a nanomaterial with a high surface area and abundant functional groups, has been utilized to immobilize laccases for efficient dye degradation in wastewater treatment [150]. The unique properties of graphene oxide enhance both the enzyme loading capacity and its operational stability on the surface, leading to more efficient and long-lasting pollutant degradation [150]. Similarly, halloysite nanotubes (HNTs) served as a support for *B. subtilis* esterase adsorption, enabling efficient dibutyl phthalate (DBP) biodegradation [151]. This non-covalent approach maintains high initial enzyme activity and allows for regeneration, making it a practical and efficient strategy for environmental bioremediation.

#### 3.1.2. Encapsulation

Encapsulation, also known as entrapment, offers a robust strategy for safeguarding enzymes and maximizing their performance in environmental applications [152]. This technique revolves around physically confining enzymes within a semi-permeable matrix. This matrix acts as a selective cage, allowing essential substrates and products to pass through while retaining the valuable enzyme molecules within its structure. While enhancing enzyme stability, encapsulation may introduce diffusional limitations and potential enzyme loss during immobilization. Conventional entrapment techniques often utilize readily available materials such as natural polymers such as alginate, chitosan, and Arabic gum, and synthetic polymers (e.g., polyacrylamide) due to their affordability, biocompatibility, and ease of processing [153,154,155,156]. Beyond these conventional polymeric matrices, biomimetic encapsulation strategies utilizing DNA and protein cages are gaining traction. DNA origami offers a highly controlled approach to constructing well-defined, intricate cages with precisely controlled pores. This exquisite control over pore size facilitates selective permeation of substrates and products while ensuring efficient enzyme retention. Notably, a three-enzyme cascade involving amylase, maltase, and glucokinase has been successfully assembled on a DNA origami triangle, demonstrating the potential for close spatial organization of enzymes for enhanced activity [157]. Additionally, a 12 nm DNA tetrahedron has been successfully employed for the encapsulation of the enzyme RNAse A, demonstrating the potential of this approach [158]. Furthermore, protein-based cages derived from viruses or ferritin represent another promising avenue for enzyme encapsulation [159,160]. These cages often possess inherent stability and can be further engineered to incorporate specific functionalities. Viral capsids, for instance, can be disassembled and reloaded with enzymes harboring attachment tags, while ferritin cages can encapsulate enzymes through well-defined electrostatic interactions [159]. This ability to engineer protein cages expands their applicability and tailorable properties for diverse environmental applications.

Biomineralized matrices composed of silica or calcium carbonate offer an extra layer of protection by forming a robust shell around the enzyme [161,162,163,164]. The encapsulation process involves meticulously mixing the enzyme solution with the chosen matrix precursor, followed by gelation, polymerization, or biomineralization [165,166]. This process culminates in the formation of stable particles or other structured forms that encapsulate the enzymes.

A key advantage of the encapsulation strategy is the exceptional protection it affords to the enzyme. Harsh environmental factors such as extreme pH or temperature fluctuations can readily deactivate free enzymes. However, entrapment shields the enzymes from these threats, significantly extending their operational lifespan and promoting remarkable stability. For example, the immobilization of *C. rugosa* lipase within calcium alginate beads resulted in a remarkable enhancement of its thermal stability [167]. Compared to the free enzyme, which exhibited only 20% residual activity after one week at 50 °C, the immobilized lipase retained a significantly higher activity of 70% under identical conditions [167]. Furthermore, the matrix can mimic the natural environment of the enzyme, creating a favorable microenvironment that preserves its native conformation and often leads to the maintenance of high activity levels. For instance, the encapsulation of lipase B from *C. antarctica* inside cowpea chlorotic mottle virus (CCMV) capsids resulted in high retention of enzymatic activity, highlighting the ability of certain matrices to maintain enzyme functionality [168]. This protection, coupled with the relative simplicity and cost-effectiveness of the approach, makes the encapsulation approach particularly well-suited for large-scale environmental remediation applications. For instance, encapsulation of laccase from *T. Versicolor* within metal–organic frameworks (MOFs) has emerged as a promising strategy for dye bioremediation [169]. The versatility and protective nature of self-encapsulation solidify its position as a cornerstone technology for various bioremediation processes.

#### 3.1.3. Covalent Immobilization

Covalent immobilization offers a powerful technique for enhancing enzyme stability and performance [170]. This method involves the formation of strong covalent bonds between the enzyme molecules and functional groups present on the support material. These support materials often possess reactive groups such as epoxides, aldehydes, isocyanates, and carbodiimides [171]. These reactive groups readily form stable and permanent linkages with specific amino acid residues on the enzyme, typically lysine or cysteine [172]. The immobilization process itself usually involves specific chemical reactions conducted under controlled conditions. This meticulous control ensures that the enzyme adopts the correct orientation and forms a secure attachment to the support.

Covalent immobilization offers a powerful combination of stability and reusability for enzymes. The strong covalent attachments formed between enzyme and support significantly reduce enzyme leaching, making it ideal for harsh environments with extreme pH, temperature, or organic solvents that would detach non-covalently bound enzymes [173]. Additionally, this method often enhances the thermal and operational stability of enzymes, allowing them to retain activity over extended periods and endure multiple reuse cycles—a significant advantage for industrial processes [174]. For instance, Laccase from *Myceliophthora thermophila*, covalently immobilized on a modified Immobead 150P carrier, retained 95% of its activity after 10 cycles at high temperature and acidic pH [175]. This superior stability compared to the free enzyme demonstrates the effectiveness of covalent immobilization for industrial applications. However, the harsh chemical conditions required for covalent bond formation can be detrimental if not carefully controlled. These conditions can lead to partial denaturation of the enzyme or improper attachment to the support, both of which significantly reduce enzymatic activity [170]. Therefore, the successful implementation of this technique relies on finding the right balance between achieving strong covalent bonds and preserving enzyme activity.

Covalent immobilization emerges as a powerful strategy to significantly enhance the stability and performance of enzymes used for micropollutant degradation. This is exemplified by a novel hollow fiber membrane bioreactor for laccase immobilization [176]. Nagatani et al. utilized radiation-induced graft polymerization (RIGP) to create a high-density polymer brush on the membrane surface containing aldehyde groups. The aldehyde groups then formed covalent bonds with laccase during immobilization, significantly improving moisture retention. The resulting bioreactor exhibited superior stability in organic media and efficient biodegradation of the pollutant bisphenol A, demonstrating the potential of this approach for industrial wastewater treatment. Similarly, the immobilization of the organophosphate-degrading enzyme OpdA on polyester fabrics exemplifies this advantage. This approach enhances the stability of OpdA and broadens its pH activity range, facilitating the efficient degradation of organophosphate pesticides [177]. The improved stability and extended operational lifespan of covalently immobilized enzymes make them ideal candidates for continuous and long-term bioremediation processes, offering a sustainable solution to the ongoing challenge of micropollutant degradation.

#### 3.1.4. Cross-Linking

Cross-linking offers a unique and advantageous approach to enzyme immobilization. This technique involves the formation of covalent bonds between enzyme molecules themselves or between enzymes and a support material [178]. The resulting structure is a stable, insoluble network with demonstrably enhanced enzyme robustness. Two prominent examples are cross-linked enzyme crystals (CLECs) and cross-linked enzyme aggregates (CLEAs) [178]. CLECs involve the initial crystallization of enzymes followed by cross-linking, while CLEAs achieve cross-linking within pre-formed enzyme aggregates, eliminating the need for prior crystallization. Common cross-linking agents, like glutaraldehyde, facilitate the formation of these stable enzyme networks. This approach yields significant advantages, such as highly stable and reusable biocatalysts. Cross-linked enzymes exhibit superior resistance to harsh conditions, including extreme pH, temperature, and organic solvents. This translates to superior mechanical stability and extended activity over multiple reuse cycles, making them highly cost-effective for industrial applications. Additionally, unlike some methods, cross-linking does not require a solid support material, potentially simplifying preparation and reducing costs.

The optimization of cross-linking protocols is paramount for successful enzyme immobilization [179]. Cross-linking agents, while facilitating the formation of desirable inter-molecular linkages between enzyme molecules, can also inadvertently lead to enzyme inactivation. Uncontrolled cross-linking reactions can result in partial denaturation of the enzyme or the formation of non-optimal cross-links, both of which significantly diminish catalytic activity. Furthermore, achieving the optimal degree of cross-linking often necessitates meticulous control of reaction parameters like time, temperature, and cross-linking agent concentration, introducing additional complexity to the immobilization process.

Cross-linked enzyme systems have been effectively used to enhance the stability and activity of enzymes for environmental applications, particularly for the degradation of micropollutants. For instance, cross-linking SulE with chitosan/gelatin significantly improved its pH and temperature tolerance compared to the free enzyme [180]. This enhanced stability allows for more efficient degradation of tribenuron-methyl and metsulfuron-methyl in soil remediation efforts. Similarly, porous-CLEAs prepared from *P. ostreatus* laccase displayed high efficiency in decolorizing and detoxifying triarylmethane and azo dyes [181]. This approach offers a promising bioremediation strategy due to the enhanced mass transfer and reusability of the immobilized enzyme, leading to effective dye removal and improved plant growth in wastewater-treated soil.

The field of cross-linking continues to evolve, offering even more tailored solutions. Researchers are exploring the use of His-rich proteins and cation-based cross-linking to form stable enzyme microparticles, as seen with *E. coli* β-galactosidase [182]. This approach mimics the structural traits of bacterial inclusion bodies and secretory amyloids, further enhancing thermal stability and reusability. By utilizing Ca^2+^ and Mg^2+^ during cross-linking, scientists can create enzymes that are highly effective for continuous and long-term bioremediation efforts. The ability of these cross-linked enzyme systems to maintain function over extended periods and harsh conditions makes them particularly valuable for addressing persistent micropollutants in various environmental contexts.

#### 3.1.5. Affinity-Based Immobilization

Conventional enzyme immobilization techniques often suffer from limitations such as random enzyme attachment and potential activity loss. Affinity tag-based immobilization offers a sophisticated and versatile alternative that leverages the power of recombinant DNA technology [26,183]. This technique involves the introduction of precisely defined peptide sequences, termed affinity tags, directly into the enzyme structure [184]. These tags exhibit exquisite selectivity for designated support materials, similar to fusion tags used in protein purification [185,186,187,188]. Well-established examples include His-tags for chelating metal matrices or biotin tags for streptavidin-coated supports [189]. Additionally, researchers have further expanded this approach by designing a diverse array of solid-binding tags. These short peptide sequences are engineered for robust binding to various materials like gold, hydroxyapatite, silica, or titania. These peptides can be fused to either the N- or C-terminus of the enzyme, enabling both single-point and multivalent immobilization strategies. For example, EctP1, a short sequence derived from the alga *Ectocarpus siliculosus*, demonstrates enhanced enzyme immobilization on both silica and titania surfaces [190]. Additionally, The incorporation of a double Sil3K peptide fusion tag to *B. subtilis* lipase A significantly augmented enzyme loading and recovered activity, culminating in a 70-fold enhancement of catalytic performance upon multivalent immobilization on diatom biosilica compared to the tag-less enzyme [27]. The exquisite selectivity of affinity tags, particularly solid-binding peptides (SBP), towards specific supports arises from a complex interplay of physicochemical interactions at the interface. This intricate network involves weak forces such as van der Waals interactions, hydrogen bonding, and electrostatic attractions. Notably, surface diffusion and potential peptide conformational changes further contribute to establishing a stable binding conformation.

Affinity tag-based immobilization offers distinct advantages beyond precise enzyme orientation. The strong and specific interaction between the tag and the support material significantly reduces enzyme leaching, a common issue with techniques relying solely on weak, non-covalent bonds. This robust binding allows for repeated enzyme use while maintaining activity. Unlike some covalent immobilization methods, affinity tags can be strategically designed to facilitate controlled release (elution) of the enzyme from the surface using mild conditions like specific salts or amino acids. For instance, proteins tagged with solid-binding peptides (SBPs) can be readily recovered for reuse using eluents such as MgCl_2_ or amino acids such as arginine or lysine, leading to high catalytic recovery [28,152,190,191]. This feature is particularly advantageous in applications requiring repeated enzyme cycles. However, some drawbacks are worth considering. The attached tag might hinder enzyme activity due to steric hindrance, and the process relies on genetic engineering to introduce the tags. This can be challenging for some enzymes and might face regulatory hurdles depending on the application.

Leveraging the key strength of precise enzyme orientation, affinity tag-based immobilization has proven remarkably effective in creating stable biocatalysts for environmental applications, particularly for the degradation of micropollutants. For example, recombinant His-tagged laccase from *Bacillus amyloliquefaciens* has been successfully immobilized on magnetic zeolitic imidazolate framework-8 (Fe_3_O_4_@ZIF-8) nanoparticles [192]. This approach demonstrates enhanced stability and reusability for degrading the dye indigo carmine. The specific binding between His-tags, unsaturated Zn atoms, and imidazole ligands on ZIF-8 surfaces ensured that the laccase remained firmly attached to the nanoparticles, maintaining 87.1% activity after 10 days of storage.

### 3.2. Techno-Economic Evaluation of Enzyme Immobilization for Bioremediation

The successful application of immobilized enzymes in bioremediation necessitates a comprehensive evaluation of associated costs. While immobilization enhances enzyme stability and reusability, the process itself incurs significant expenditures on raw materials, equipment, and labor. Natural polymers such as alginate offer cost-effective options, whereas advanced supports such as metal–organic frameworks demand substantial investments. Moreover, operational expenses, including energy consumption and waste management, must be factored into the overall economic assessment.

To mitigate these costs, the exploration of alternative support materials is imperative. Agricultural residues, including lignocellulosic materials, biochar, and eggshell-derived materials, present promising opportunities [193,194]. These materials are abundant, inexpensive, and often underutilized, leading to environmental problems when improperly disposed of. Converting these wastes into porous supports for enzyme immobilization not only reduces overall costs but also addresses environmental issues. Furthermore, optimizing immobilization conditions and developing efficient recovery methods can contribute to reducing operational expenses.

A holistic techno-economic analysis, encompassing raw material costs, energy consumption, labor, equipment, and process optimization, is essential for evaluating the economic viability of enzyme immobilization on a large scale. By carefully considering these factors, it is possible to identify cost-effective strategies and maximize the return on investment for bioremediation applications.

## 4. Applications of Immobilized Microbial Enzymes for Multipollutant Mitigation

The proliferation of various pollutants, including pharmaceuticals, pesticides, and industrial chemicals, in the environment poses significant risks to ecosystems and human health. Traditional wastewater treatment methods have limitations in removing these contaminants entirely, leading to their accumulation in water bodies and soil. Therefore, immobilized microbial enzymes offer a promising solution for the bioremediation of these pollutants due to their enhanced stability, reusability, and specificity. By anchoring enzymes onto solid supports, their catalytic activity can be maintained over extended periods, making them highly effective for continuous pollutant degradation processes. In this section, we will explore the application of immobilized microbial enzymes for mitigating multiple pollutants (Table 1).

### 4.1. Immobilized Microbial Enzymes for Mitigating Pharmaceutical Pollutants

The ubiquitous presence of pharmaceutical contaminants, encompassing antibiotics, hormones, analgesics, and a diverse array of prescription and over-the-counter medications, is increasingly documented across various environmental matrices. These pollutants infiltrate the environment through a multitude of pathways, including improper disposal practices, excretion from treated humans and livestock, and effluent discharge from pharmaceutical manufacturing facilities. The widespread occurrence of antibiotics in aquatic ecosystems is particularly concerning, as it can contribute to the emergence and dissemination of antibiotic-resistant bacteria, posing a significant public health threat. Additionally, hormones, even at minute concentrations, can disrupt the endocrine systems of aquatic organisms, leading to detrimental effects on reproduction and development. Furthermore, other pharmaceutical compounds can exert toxic effects on non-target species, culminating in biodiversity loss and a disruption of ecosystem equilibrium. The recalcitrance of these pollutants necessitates the exploration of advanced treatment methodologies to effectively mitigate their environmental impact.

Immobilized microbial enzymes have demonstrated significant potential in degrading various pharmaceutical pollutants, offering a sustainable approach to mitigating their environmental impact. One promising approach for mitigating antibiotic pollution involves erythromycin esterase type II (EreB), an enzyme specifically designed to degrade erythromycin, a common macrolide antibiotic. However, limitations in EreB’s stability and reusability hinder its widespread application. Accordingly, recent research has explored several innovative strategies for immobilizing EreB esterase, aiming to enhance its effectiveness in eliminating erythromycin. The first study investigates the efficacy of enzymatic membrane reactors (EMRs) [195]. The immobilized EreB esterase, obtained from a genetically modified *E. coli* strain, onto the EMR by adsorption approach displayed remarkable stability. The immobilized enzyme continuously degraded erythromycin at a rate of 15.8 mg/h for 100 h, demonstrating its long-term efficacy. The second study explored palygorskite, a naturally occurring clay mineral, as a carrier for EreB immobilization [196]. The resulting EreB@modified palygorskite composite exhibited enhanced stability across a broader range of temperatures and pH values, coupled with increased enzymatic activity. Importantly, it retained 45% activity over 10 reuses and effectively degraded erythromycin in polluted water 20 mg L^−1^ within 300 min. Additionally, Ni et al. investigated the use of Cu-BTC, a synthetic metal–organic framework, as a platform for EreB immobilization [197]. The EreB@Cu-BTC composite demonstrated improved stability towards heat and alkaline conditions, along with a heightened affinity for its target substrate, erythromycin. Notably, the immobilized enzyme maintained 57.12% over 10 cycles and effectively degraded erythromycin in wastewater, eliminating its antibacterial activity. These studies showcase the transformative potential of enzyme immobilization techniques. By substantially enhancing the stability, reusability, and catalytic efficiency of EreB esterase, these approaches offer a promising foundation for the development of environmentally sound and practical solutions to antibiotic contamination in water. These advancements contribute to improved ecosystem health and public safety.

Laccase-based biocatalysts hold significant potential for mitigating antibiotic pollution in water. Shao et al. investigated a novel strategy for mitigating antibiotic pollution—the immobilization of laccase (Lac) from *T. versicolor* onto hollow mesoporous carbon spheres (HMCs) [198]. This approach leverages the enzyme’s ability to degrade antibiotics while addressing limitations associated with free Lac. The researchers explored two immobilization techniques: covalent interaction and physical adsorption. Through successful HMC synthesis and modification, they achieved efficient Lac immobilization with a maximum loading capacity of 835 mg/g. Notably, the immobilized Lac exhibited significantly improved stability across various parameters (temperature, pH, and storage) and reusability compared to the free enzyme. Furthermore, it demonstrated excellent removal efficiency for tetracycline hydrochloride (TCH) and ciprofloxacin hydrochloride (CPH) antibiotics, achieving removal rates of 99.4% and 96.9%, respectively, in the presence of a redox mediator. This high efficiency is likely attributed to the synergistic effect of HMC adsorption and Lac-mediated degradation. These findings suggest that the immobilization of laccase on HMCs holds promise for bioremediation of a broader spectrum of antibiotic pollutants in environmental applications.

**Figure 5:**
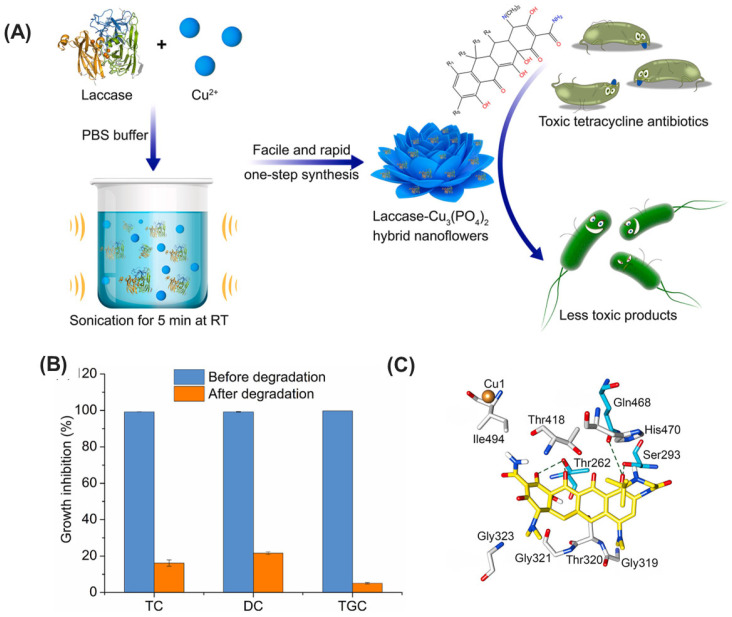
Application of Lac-hNFs for tetracycline degradation. (**A**) Schematic illustration of laccase-inorganic hybrid nanoflower (Lac-hNF) preparation for tetracycline (TC) degradation. (**B**) Bacterial growth inhibition by TCs was assessed before and after Lac-hNF treatment. Lac-hNF treatment reduced *E. coli*’s susceptibility to TCs. (**C**) Depiction of the binding interactions between laccase and tigecycline (TGC), as revealed by molecular docking simulations. (**A**–**C**): Reproduced with permission from [199], Copyright 2023 Elsevier Inc.

Han et al. investigated the application of immobilized laccase in the form of laccase-inorganic hybrid nanoflowers (Lac-hNFs) for the degradation of tetracycline antibiotics (TCs), an area previously lacking extensive exploration (Figure 5) [199]. Lac-hNFs were rapidly fabricated using a sonication method and demonstrated significantly enhanced thermal and storage stability compared to the free enzyme. Notably, Lac-hNFs exhibited superior performance in degrading a variety of TCs, including tigecycline, which has not been previously reported for laccase treatment. When employed in conjunction with acetosyringone as a mediator, Lac-hNFs efficiently removed over 79% of the TCs within an hour. Furthermore, Lac-hNFs displayed remarkable reusability, maintaining a high degradation capacity after multiple cycles. Importantly, the study confirmed a significant reduction in TC toxicity following Lac-hNF treatment, suggesting its potential application in environmental remediation efforts. The research also identified the main transformation products of the TCs and proposed degradation pathways. Additionally, molecular docking simulations provided valuable insights into the interaction between laccase and TCs. All TCs docked into a similar pocket near the enzyme’s copper center, with tigecycline exhibiting the strongest binding affinity, which aligns with the observed degradation efficiencies. This finding suggests potential avenues for future enzyme engineering endeavors aimed at further enhancing laccase’s efficiency in antibiotic degradation. Overall, this work highlights the significant impact of Lac-hNFs in immobilizing laccase for the effective removal of antibiotic pollutants from the environment.

Expanding the capabilities of immobilized laccase for antibiotic remediation has been the focus of recent research. One study explored magnetic cross-linked enzyme aggregates (M-CLEAs) as a carrier for laccase derived from *Cerrena unicolor* [200]. The resultant immobilized laccase exhibited exceptional efficacy, particularly for tetracycline (TC) and oxytetracycline (OTC) degradation, achieving complete removal of 100 μg/mL TC within 48 h under optimal conditions (pH 6, 25 °C) without requiring a redox mediator. Notably, laccase treatment significantly reduced the antimicrobial activity of both TC and OTC, suggesting a potential mechanism for its role in antibiotic breakdown. These findings highlight M-CLEAs as a promising and environmentally friendly approach for antibiotic removal in water treatment, solidifying the effectiveness of immobilized laccase in bioremediation applications. Complementing this approach, Zou et al. investigated the potential of laccase immobilized on magnetic biochar (LC-MBC) for removing a different class of pollutants: quinolone antibiotics (norfloxacin, enrofloxacin, and moxifloxacin) from wastewater [201]. Compared to free laccase, LC-MBC exhibited significantly higher removal efficiencies for these target antibiotics (up to 93.7%). This improved performance is attributed to the synergistic effect of adsorption by the magnetic biochar and degradation by the immobilized laccase.

Pharmaceutical contamination in aquatic ecosystems extends beyond antibiotics, encompassing endocrine-disrupting compounds (EDCs) such as hormones. Even at trace levels, these hormones can wreak havoc on the endocrine systems of aquatic organisms, leading to reproductive and developmental issues. Conventional water treatment processes often prove inadequate in removing these potent EDCs, necessitating the exploration of alternative bioremediation strategies. Research suggests significant promise in utilizing microbial enzymes specifically tailored to target and degrade hormones present in wastewater. These enzymes offer a targeted and environmentally friendly approach compared to traditional methods. However, further optimization is required to ensure their efficacy in real-world applications. This includes enhancing their stability and reusability within the complex and variable composition of wastewater environments.

Lacerda et al. explored a promising approach for mitigating pharmaceutical pollutants in wastewater: immobilized laccase from *P. ostreatus*. This enzyme targets 17α-ethinylestradiol (EE2), a prevalent and concerning endocrine-disrupting compound (EDC) [221]. The researchers investigated *Luffa cylindrica* fibers as a carrier to immobilize the laccase, enhancing its resilience towards variations in pH and temperature—a critical factor for real-world wastewater treatment applications. Notably, at an optimal pH of 5, immobilized laccase achieved a significantly higher removal rate (64.28%) of EE2 compared to free laccase (44.23%). This 20% improvement highlights the potential of immobilization for boosting enzyme performance in EDC degradation. This study highlights the potential of immobilized laccase from *P. ostreatus* as a viable bioremediation tool for EE2 removal. Although further optimization is necessary to maximize efficiency, these findings provide a foundation for developing eco-friendly and sustainable approaches to address pharmaceutical pollution in wastewater treatment.

A novel biocatalytic system utilizing immobilized laccase from *T. versicolor* on 3D printed polylactide (PLA) scaffolds via physical adsorption has demonstrated significant potential for the removal of hormonal contaminants from wastewater [202]. The best performance for the immobilized enzyme was achieved at pH 5, with an enzyme concentration of 5 mg/mL and an immobilization duration of 24 h. Although immobilization slightly reduced the reactivity of laccase, it led to substantial improvements in its chemical and thermal stability. After 20 days of storage at 4 °C, the immobilized laccase retained 80% of its initial activity, compared to only 35% for the free enzyme. In practical applications, the immobilized laccase on 3D printed PLA scaffolds achieved a 10% improvement in the removal of estrogens from real wastewater compared to the free enzyme, and it exhibited significant reusability potential. The biocatalytic system removed approximately 40% of estradiol (E2) and ethinylestradiol (EE2) from municipal wastewater.

A recent study presents a novel biocatalytic approach for the degradation of estrogenic endocrine-disrupting compounds (EDCs) in wastewater [222]. This environmentally friendly method utilizes immobilized laccase enzymes derived from *Lysinibacillus* sp. BP1 and BP2 bacteria. The research employed a central composite design to optimize key parameters—pH, inoculum size, and copper concentration—for enhanced laccase activity and estrogen removal efficiency. The resulting biocatalysts consisted of laccase immobilized on either glass beads or silver-impregnated clay granules (SICG). Notably, the immobilized enzymes displayed enhanced storage stability and reusability, crucial factors for long-term applications. Furthermore, the immobilized enzymes achieved impressive estrogen removal capabilities. Within a 24-h timeframe, they achieved complete removal (100%) of 17β-estradiol (E2) and over 90% removal of both estrone (E1) and 17α-ethynylestradiol (EE2). This high efficiency suggests their potential for large-scale implementation in benchtop bioreactors for wastewater treatment. While both glass beads and SICG biocatalysts demonstrated comparable estrogen removal rates (ranging from 95% to 100%), glass beads emerged as the preferable carrier material due to their superior recyclability and stability under various conditions. SICG, however, offers an intriguing alternative due to its potential antibacterial properties and cost-effectiveness, warranting further exploration for large-scale wastewater treatment applications.

Emerging pharmaceutical contaminants such as ibuprofen (IBF), diclofenac (DCF), and carbamazepine pose a growing challenge for conventional wastewater treatment processes. Bhardwaj et al. investigate the efficacy of immobilized laccase enzymes for the removal of these pollutants [223]. A laccase derived from *Alcaligenes faecalis* XF1 was immobilized onto a copper-based metal–organic framework (CuBTC), resulting in a high protein loading capacity of approximately 67.48%. Notably, even after 14 days of storage at 4 °C, the immobilized laccase retained a remarkable 86% of its activity, demonstrating superior stability compared to the free enzyme. Importantly, laccase@CuBTC displayed exceptional efficiency in degrading IBF and DCF. Without requiring additional mediators, it achieved around 95% DCF removal within 60 min and nearly 96% IBF degradation in just 3 h. In comparison, the free enzyme exhibited significantly slower degradation rates. These findings suggest that laccase@CuBTC offers a promising and more stable, reusable, and storage-friendly method for the biocatalytic removal of pharmaceutical contaminants like IBF and DCF from wastewater streams.

In the face of escalating pharmaceutical contamination that threatened to overwhelm conventional wastewater treatment methods, Touahar et al. developed robust biocatalysts, termed combi-CLEAs, through the cross-linking of a potent enzymatic cocktail sourced from distinct microorganisms: laccase from *T. versicolor*, versatile peroxidase from *Bjerkandera adusta*, and glucose oxidase from *A. niger* [203]. Notably, these combi-CLEAs retained significant activity (around 30–40% for each enzyme) even after immobilization, showcasing their efficiency. Compared to individual enzymes, the combi-CLEA demonstrated a wider range of degradation capabilities, particularly effective against commonly encountered pharmaceuticals such as acetaminophen, naproxen, mefenamic acid, diclofenac, and indometacin. This enhanced activity is attributed to the synergistic action of all three enzymes, with versatile peroxidase from *Bjerkandera adusta* playing a key role in broadening the degradation spectrum. The study further explored the application of combi-CLEAs in treating municipal wastewater effluents. These initial promising results warrant further investigation into optimizing combi-CLEA production and expanding their use for the efficient and eco-friendly removal of a wider range of pharmaceutical contaminants from wastewater streams.

Overall, the application of immobilized microbial enzymes for the degradation of pharmaceutical pollutants offers a robust and efficient approach to reducing their environmental footprint. By targeting specific drug classes and utilizing tailored enzymatic solutions, it is possible to enhance the effectiveness of bioremediation efforts and protect both ecosystems and human health from the adverse effects of these persistent pollutants.

### 4.2. Dyes and Pigments as Environmental Micropollutants

Emerging as a significant environmental concern are dyes and pigments, particularly those employed in the textile and manufacturing industries. Their inherent stability, toxicity, and recalcitrance (resistance to degradation) pose a serious threat to ecological well-being. Untreated wastewater discharges containing these pollutants can have a devastating impact on water quality and aquatic life. Two major classes of concern are azo dyes and synthetic dyes, both ubiquitous across various industrial applications. Fortunately, immobilized enzymes offer a promising and sustainable approach to the degradation of dyes and pigments in wastewater treatment.

Immobilized manganese peroxidase (MnP) has shown great promise in treating textile wastewater, a major source of dye pollution. One study investigated the immobilization of MnP from the fungus *Aspergillus flavus* onto iron oxide nanoparticles [204]. The immobilized MnP displayed enhanced thermal stability, functioning effectively across a wider range of pH and temperatures (optimal pH 5.0, 50 °C). It also exhibited improved catalytic activity, achieving complete decolorization of Direct Red 31 and significantly improved (92%) decolorization of Acid Black 234 compared to the free enzyme. Additionally, the magnetic properties of the nanoparticles facilitated easy separation and reuse, promoting a more sustainable and cost-effective wastewater treatment solution. Another study addressed a critical limitation of MnP—its dependence on Mn^2+^ ions for activity [205]. To overcome this hurdle, Vizcarra et al. developed a novel approach involving the co-immobilization of MnP and Mn^2+^ ions within silica gels. This strategy significantly boosted MnP activity (4–5 times higher) compared to MnP immobilized alone. The presence of Mn^3+^ within the gel confirmed the continued functionality of the immobilized Mn^2+^ ions in electron shuttling, an essential process for MnP activity. This enhancement translated to superior performance in dye removal tests, with the co-immobilized system achieving 2–4 times higher removal rates. This innovation paves the way for broader MnP applications by enabling its effective utilization even in environments lacking Mn^2+^ ions.

The potential of MnP extends beyond wastewater treatment. Researchers have developed a novel biosensor utilizing MnP immobilized onto a carbon-felt electrode [224]. This sensor exploits the inhibitory effect of certain dyes on MnP activity. By measuring changes in electrical current as dye concentration increases, the sensor can differentiate between dyes with varying toxicity levels. For instance, Reactive Red 195 (RR195) exhibits an inhibitory effect, while Reactive Blue 221 (RB221) does not, reflecting their relative toxicity. This concept was validated through separate experiments, highlighting the sensor’s potential for dye monitoring and toxicity assessment. Furthermore, the sensor boasts several advantages, including low cost, disposability, durability, a low detection threshold, and minimal interference from external substances. These advancements position MnP as a cornerstone for revolutionizing wastewater treatment processes and promoting eco-friendly industrial practices, with ongoing research unlocking its full potential in environmental remediation efforts.

Laccase, another potent enzyme, has been extensively studied for its ability to degrade a wide variety of dyes, making it a valuable tool in environmental remediation, especially for textile wastewater treatment. Several innovative immobilization methods have been developed that significantly enhance the enzyme’s performance and applicability. One novel approach involves the co-immobilization of laccase from *Aspergillus* sp. with a mediator (ABTS) onto a metal–organic framework (MOF) grown on PET fibers [174]. This method improves the stability of immobilized laccase in acidic environments and enables the degradation of previously resistant dyes such as crystal violet and malachite green. The co-immobilized laccase-mediator system (LMS) demonstrated significantly higher dye removal rates compared to free laccase, achieving a 58.8% removal of crystal violet within 24 h, which is seven times higher than that of free laccase. Additionally, both laccase and ABTS can be recycled, minimizing waste and promoting sustainability.

**Figure 6:**
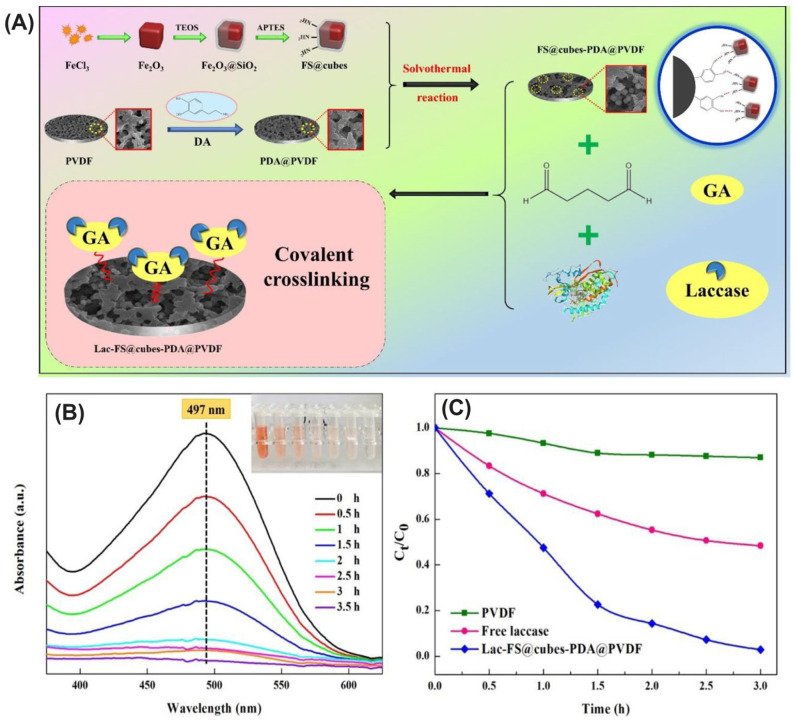
Biocatalytic membrane for efficient Congo Red removal. (**A**) Schematic illustration of the Lac-FS@cubes-PDA@PVDF membrane fabrication process. (**B**) UV-vis spectra depicting Congo Red removal by the Lac-FS@cubes-PDA@PVDF membrane over time (inset: illustration of the color change during dye removal). (**C**) Comparison of Congo Red decoloration efficiency between the pristine PVDF membrane, free laccase, and the Lac-FS@cubes-PDA@PVDF membrane. (**A**–**C**): Reproduced with permission from [225], Copyright 2020 Elsevier B.V.

Another innovative method addresses the challenge of removing toxic azo dyes by developing a biocatalytic membrane using a robust polyvinylidene fluoride (PVDF) base [225]. Researchers created a hybrid bio-inorganic structure on the membrane’s surface through a multi-step process: coating the PVDF membrane with polydopamine (PDA), attaching specially designed iron oxide cubes modified with silica (Fe_2_O_3_@SiO_2_) and a coupling agent (APTES), and finally immobilizing laccase onto this structure (Figure 6). The resulting Lac-FS@cubes-PDA@PVDF membrane achieved impressive results, efficiently removing the model azo dye Congo Red with 97.1% efficiency, displaying excellent storage stability, and maintaining high reusability (over 75% removal efficiency after five cycles). This study underscores the potential of biocatalytic membranes with hybrid bio-inorganic structures for wastewater treatment, presenting a simple, efficient, and reusable approach for laccase immobilization.

A novel co-immobilization strategy using polyethyleneimine (PEI)-induced biomimetic mineralization was investigated to enhance the application of recombinant *B. subilits* laccase for dye biodegradation [206]. Li et al. created two carrier materials, PEI@CaP and (PEI + ABTS)@CaP, for the co-immobilization of laccase and a mediator molecule (ABTS). The negative charge of laccase facilitated its immobilization onto the carriers through electrostatic adsorption. Both immobilized laccase preparations [Lac-PEI@CaP and Lac-(PEI + ABTS)@CaP] exhibited significant improvements compared to the free enzyme, including high activity recovery, enhanced pH tolerance, and improved storage stability. Notably, Lac-(PEI + ABTS)@CaP offered a distinct advantage by co-immobilizing ABTS alongside laccase within the carrier. This proximity effect between the enzyme and mediator molecules resulted in superior malachite green (MG) degradation capability compared to systems using free ABTS. Furthermore, Lac-(PEI + ABTS)@CaP demonstrated reusability, retaining over 80% of its MG degradation activity after three cycles. This research underscores the significant impact of laccase immobilization using PEI-induced biomimetic mineralization. This approach presents a promising strategy for developing robust, reusable biocatalysts for efficient dye removal in wastewater treatment applications. While some challenges regarding enzyme and mediator leakage during operation require further investigation, this study represents a significant advancement in utilizing immobilized laccase for environmental remediation efforts.

A further advancement in laccase immobilization involves using metal–organic frameworks (MOFs) and hydrogels to overcome the limitations of the laccase-mediator system (LMS) [226]. Researchers created a PVA-Lac@Cu/ZIFs biocatalyst by encapsulating laccase with copper-based MOFs within a poly(vinyl alcohol) (PVA) hydrogel matrix. This approach resulted in a highly stable biocatalyst that efficiently degraded malachite green dye (100% within 4 h). Additionally, both the laccase and mediator were successfully recycled and reused for multiple dye degradation cycles, maintaining significant activity. This study demonstrates the potential of MOFs and hydrogels for co-immobilizing LMS, offering enhanced stability, reusability, and efficiency in dye removal. This approach holds promise for developing sustainable and practical solutions to wastewater treatment challenges.

Tyrosinase, a well-known oxidoreductase, is effective in removing hazardous and toxic dyes and phenolic compounds, which are significant environmental pollutants. A recent study successfully immobilized tyrosinase from *Agaricus bisporus* onto silver-coated Fe_3_O_4_ nanoparticles through covalent bonding using EDC/NHS chemistry. This engineered biocatalyst demonstrated high efficiency, practicality, and reusability in pollutant removal applications [207]. The immobilized tyrosinase amount was calculated to be 216.6 ± 1.250 mg per gram of nanoparticles. Immobilization increased the enzyme’s substrate affinity by 1.4-fold and preserved 48.9% of its original activity after 6 reuses. After 84 days of storage, the residual activity of the immobilized enzyme was 68.3%, compared to 45.8% for the free enzyme. The immobilized tyrosinase exhibited high efficacy in the removal of both azo dyes and phenolic compounds from aqueous solutions. It achieved decolorization rates of 95.0% and 36.9% for Congo Red and Reactive Green 19, respectively. Furthermore, the biocatalyst demonstrated significant removal capabilities for a range of phenolic compounds, including 87.8% removal of 4-chlorophenol and 92.3% removal of phenyl acetate. Moreover, the electrochemical properties of immobilized tyrosinase were characterized for its potential application as a catechol biosensor using cyclic voltammetry. This research contributes to the development of environmentally benign bioremediation strategies for the removal of azo dyes and phenolic contaminants from wastewater.

### 4.3. Pesticides as Environmental Micropollutants

The widespread application of pesticides, encompassing insecticides, herbicides, and fungicides, has undeniably bolstered agricultural productivity. However, their persistence in soil and water poses a significant environmental threat. Bioaccumulation within ecosystems and potential harm to non-target organisms, including humans, necessitate alternative remediation strategies. Immobilized enzymes emerge as a promising green technology. Several immobilized enzyme biocatalysts are specifically designed to degrade pesticide molecules into less harmful compounds, effectively reducing their environmental persistence and toxicity. This approach presents a sustainable and targeted solution for mitigating pesticide pollution.

Organophosphate (OP) insecticides, including malathion, chlorpyrifos, and paraoxon, offer significant pest control benefits. However, their persistence in the environment and potent neurotoxicity raises concerns for non-target organisms and human health. Thankfully, research has yielded a promising solution: biocatalyst enzymes specifically designed to degrade these toxic compounds. To tackle malathion pesticide pollution, researchers engineered a novel enzyme (EstM160K) derived from *Geobacillus uzenensis* [208]. This enzyme exhibits significantly improved stability at high temperatures (70 °C) due to a strategic mutation and immobilization process. The resulting biocatalyst, lx-EstM160K (esterase immobilized on epoxy resin lx-105s), effectively degrades malathion, achieving a removal rate of 95.8% at moderate concentrations (20 mg/L). Additionally, lx-EstM160K demonstrates reusability, maintaining good activity in wastewater treatment applications. Another study tackled the challenge of chlorpyrifos degradation using immobilized laccase from *Bacillus* sp. onto magnetic nanoparticles [227]. They optimized the process to achieve near-complete recovery of enzyme activity. The resulting immobilized laccase boasted several improvements: greater stability (lasting 100 h), tolerance to alkaline pH, higher temperatures, and, importantly, the ability to effectively degrade the pesticide chlorpyrifos. In further study tackling the challenge of degrading the toxic pesticide paraoxon, researchers have developed a highly effective method utilizing immobilized phosphotriesterase (PTE) enzyme from *Sulfolobus solfataricus* within a specialized biocatalytic membrane [209]. Their key innovation centers on incorporating either cationic (CTAB) or anionic (SDS) surfactants with the immobilized PTE. This approach significantly enhances PTE activity: free enzyme activity increased up to 90% with both surfactants, likely due to CTAB-induced structural changes or SDS-facilitated increased substrate affinity. Although the improvement in immobilized enzyme activity was slightly lower due to reduced flexibility, the resulting biocatalytic membranes exhibit substantial advantages. These membranes boast doubled specific activity compared to the system without surfactants, maintain activity through multiple reaction cycles with surfactant replenishment, and achieve a remarkable 96% paraoxon conversion rate in a biocatalytic reactor, requiring only one-third the time reported in previous methods. This study highlights the potential of immobilized PTE enzymes combined with surfactants for creating highly active and stable biocatalytic membranes. This approach represents a significant leap forward in degrading organophosphate pesticides and overcoming limitations that previously hindered the large-scale application of this technology.

**Figure 7:**
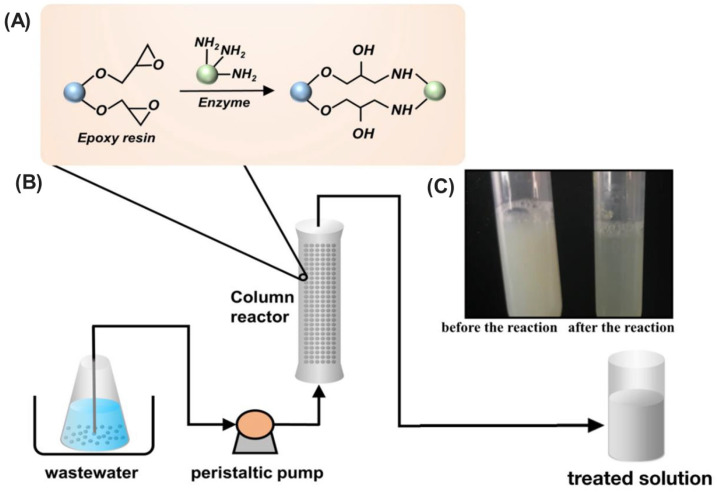
Continuous bifenthrin degradation using a packed-bed bioreactor with immobilized esterase. (**A**) The immobilization process of EstM160K, an enzyme derived from *Geobacillus uzenensis*, onto epoxy resin LX-105S. (**B**) The experimental setup of the reactor highlights the use of the immobilized LX-EstM160K biocatalyst for bifenthrin wastewater treatment. (**C**) Visual demonstration of the effectiveness of the process, comparing samples before and after treatment with LX-EstM160K in the reactor. (**A**–**C**): Reproduced with permission from [208], Copyright 2020 MDPI (CC BY 4.0).

Pyrethroid insecticides, such as fenpropathrin, cypermethrin, permethrin, and bifenthrin, provide significant benefits in pest control. However, their persistence in the environment and potent neurotoxic effects raise concerns regarding their impact on non-target organisms and human health. Pyrethroids disrupt the insect nervous system by targeting sodium channels, leading to paralysis and, ultimately, insect death. Recent studies explore a novel approach for pyrethroid degradation utilizing immobilized esterase enzymes. Zong et al. identified a novel gene (est882) encoding an effective esterase enzyme (Est882) capable of efficiently degrading pyrethroids such as fenpropathrin, cypermethrin, and permethrin, achieving over 80% degradation within 30 min [228]. To further enhance stability and broaden its applicability, Est882 was immobilized, resulting in a significant improvement in its tolerance and effectiveness across various environmental conditions. Further supporting the potential of immobilized esterases for pyrethroid remediation, another study investigated lx-EstM160K, an immobilized esterase derived from *Geobacillus uzenensis* [208]. Notably, lx-EstM160K exhibited exceptional degradation efficiency (90.4%) for bifenthrin at high concentrations (500 mg/L) within a reactor system. Additionally, lx-EstM160K demonstrated remarkable operational stability, retaining 72% of its initial activity after ten continuous cycles of bifenthrin degradation (Figure 7). These findings strongly suggest the promising potential of immobilized esterases for bioremediation efforts in pyrethroid-contaminated environments.

Carbamates, a class of fungicides, insecticides, and herbicides derived from carbamic acid, offer a lower human health risk compared to organophosphate pesticides [229]. Researchers investigated a novel approach for wastewater treatment that leverages immobilized laccase enzymes to target these carbamate pollutants [230]. This method involved attaching laccase enzymes, isolated from the fungus *T. versicolor*, onto specially designed microbeads containing carbonate or epoxy groups. The immobilization process significantly enhanced enzyme performance. Compared to the free enzyme, the immobilized laccase achieved near-complete biodegradation of the model carbamate pesticide, carbaryl, in the presence of a mediator. Additionally, the immobilized enzyme exhibited broader tolerance for variations in pH and temperature, along with improved storage stability. When tested in a fluidized bed reactor for a day, the immobilized enzyme displayed minimal activity loss for carbaryl, suggesting excellent reusability. This study highlights the potential of immobilized laccase on microbeads as a promising strategy for effectively removing carbamate micropollutants from wastewater treatment. This approach offers a three-pronged benefit: improved degradation efficiency, enhanced enzyme stability, and good reusability.

Organochlorines, a class of pesticides including dichlorodiphenyltrichloroethane (DDT) and dichlorophen, are well known for their long-term persistence in the environment and potential to bioaccumulate, posing significant ecological and health risks. Researchers have developed a promising solution for environmental remediation, a novel biocatalyst specifically targeting dichlorophen degradation: laccase-MSU-F [210]. This innovative material is a hybrid nanomaterial combining laccase enzyme, derived from the fungus *Coriollopsis gallica*, with mesoporous synthetic silica foam (MSU-F). Laccase-MSU-F effectively degraded dichlorophen, potentially through chlorine removal or the formation of larger polymer structures. This degradation offered a two-pronged benefit: it significantly reduced the pesticide’s genotoxicity and apoptotic effects in cell studies, indicating lower cellular damage. Additionally, the study suggested that the breakdown products might have a lower affinity for binding to steroid hormone receptors, potentially reducing the risk of endocrine disruption associated with the original pesticide. Overall, laccase-MSU-F demonstrates great promise for environmental remediation by degrading dichlorophen and mitigating its harmful effects, with potential for broader applications beyond environmental uses.

In a promising development for DDT remediation, Salem et al. created a powerful biocatalyst using covalently immobilized ligninolytic enzymes [211]. They first produced a cocktail of these enzymes—laccase, aryl alcohol oxidase, lignin peroxidase, and manganese peroxidase—from the fungus *P. ostreatus*. Following partial purification, the enzymes underwent covalent immobilization onto nano-silica particles using glutaraldehyde. This process significantly enhanced the enzymes’ stability and reusability. The resulting biocatalyst displayed impressive activity and stability across a wide range of pH (4–9) and temperature (20–55 °C). Most importantly, it effectively degraded the pesticide p,p′-DDT, achieving complete elimination within just 12 h under specific conditions (pH 5 and 30 °C). Additionally, the biocatalyst maintained good activity even after multiple reuse cycles. These findings highlight the promise of covalently immobilized ligninolytic enzymes from *P. ostreatus* on nano-silica as a cost-effective and reusable strategy for DDT degradation and potentially broader environmental remediation applications. The success of these immobilized biocatalysts demonstrates their potential impact on environmental remediation, offering a viable approach to mitigate the harmful effects of persistent organochlorine pollutants and potentially extending their application to broader environmental challenges.

**Figure 8:**
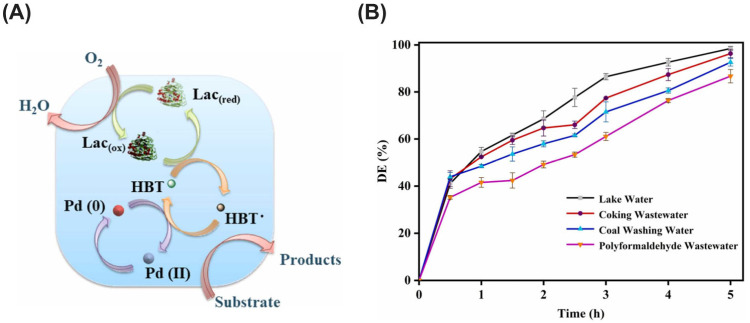
Lac-HBT-Pd/BC biocatalyst for atrazine degradation. (**A**) Proposed degradation pathway of atrazine (ATZ) by the Lac-HBT-Pd/BC biocatalyst. (**B**) The degradation efficiency of ATZ in various water environments by Lac-HBT-Pd/BC. (**A**,**B**): Reproduced with permission from [231], Copyright 2024 Elsevier B.V.

The widespread use of herbicides, such as atrazine and mesotrione, for weed control poses a significant environmental challenge due to their persistence in soil and water, leading to adverse effects on plant and aquatic life. To address this growing concern, researchers are actively exploring the development of microbial enzyme biocatalysts capable of degrading these persistent pollutants. One promising approach involves the use of co-immobilized enzyme membrane composites [231]. A recent study demonstrated the effectiveness of Lac-HBT-Pd/BC, a novel biocatalyst material integrating three key components: laccase enzyme for degradation, 1-hydroxybenzotriazole (HBT) as a mediator molecule, and palladium (Pd) metal (Figure 8). All three elements were co-immobilized onto a functionalized bacterial cellulose (BC) carrier. This innovative design enabled Lac-HBT-Pd/BC to achieve exceptional performance in atrazine degradation within water treatment systems. The biocatalyst achieved complete removal of atrazine within 5 h under mild ambient conditions. Notably, it also exhibited impressive efficiency in degrading toxic intermediate byproducts formed during atrazine breakdown (around 85% removal), resulting in a “deep degradation” process. The inclusion of Pd metal played a crucial role by enhancing the stability of mediator radicals and boosting the overall catalytic activity of the biocatalyst. Furthermore, Lac-HBT-Pd/BC demonstrated excellent reusability and adaptability to various water qualities, signifying its potential for practical applications in biocatalytic water treatment. This study underscores the significant potential of co-immobilized enzyme membrane composites for effective and sustainable remediation of persistent pollutants like atrazine.

Another research effort investigated the application of immobilized laccase enzymes for the degradation of mesotrione (MES), an emerging environmental pollutant. Yue et al. employed granular zinc oxide (G-ZnO) as a carrier material for laccase immobilization (G-ZnO@Lac) [232]. This approach resulted in a significant enhancement of enzyme stability. The immobilized laccase, G-ZnO@Lac, retained over 50% activity after 28 days compared to a mere 12% for the free enzyme. Furthermore, G-ZnO@Lac exhibited improved thermal stability, acid–base stability, and reusability. Importantly, the immobilized laccase also demonstrated efficacy in MES degradation, achieving a 73.25% degradation rate under optimized conditions. These findings highlight the transformative impact of immobilization on laccase performance. The resulting G-ZnO@Lac system presents a promising and practical strategy for MES degradation and potentially broader applications in environmental remediation due to its enhanced stability, reusability, and overall effectiveness.

Overall, the application of immobilized microbial enzymes for the degradation of pesticide pollutants provides a promising approach to addressing the environmental and health impacts of these persistent contaminants. By harnessing the specificity and efficiency of enzymatic degradation, it is possible to reduce the persistence and toxicity of pesticides in the environment, contributing to the protection of ecosystems and human health.

### 4.4. Degradation of Microplastics with Immobilized Enzymes

Microplastics, tiny fragments of plastic such as polyethylene terephthalate (PET), polystyrene (PS), and polypropylene (PP) that are less than 5 mm in size, contaminate environments due to debris breakdown or direct manufacturing. These pollutants pose significant threats through ingestion and chemical leaching. Fortunately, specially designed immobilized enzymes offer a sustainable solution for mitigating this microplastic pollution by breaking down these persistent pollutants.

In a significant advancement for bioremediation of plastic pollutants, researchers immobilized polyethylene terephthalate hydrolase (PETase) onto cobalt phosphate (Co_3_(PO_4_)_2_) nanostructures via biomimetic mineralization [212]. This approach significantly enhanced PETase performance. The high surface area of the Co_3_(PO_4_)_2_ nanocarriers facilitated increased enzyme loading, leading to superior catalytic activity. Additionally, the immobilized PETase (PETase@Co_3_(PO_4_)_2_) exhibited improved stability with broader temperature and pH tolerance compared to the free enzyme. This translated to a longer lifespan and enhanced degradation efficiency. Notably, PETase@Co_3_(PO_4_)_2_ demonstrated significantly higher terephthalic acid (TPA) production, a key PET breakdown product, and retained substantial activity even after extended use, highlighting its reusability. Overall, this study highlights the potential of biomimetic mineralization as a promising approach for enzyme immobilization, offering a pathway toward more effective and sustainable strategies for combating PET plastic pollution.

**Figure 9:**
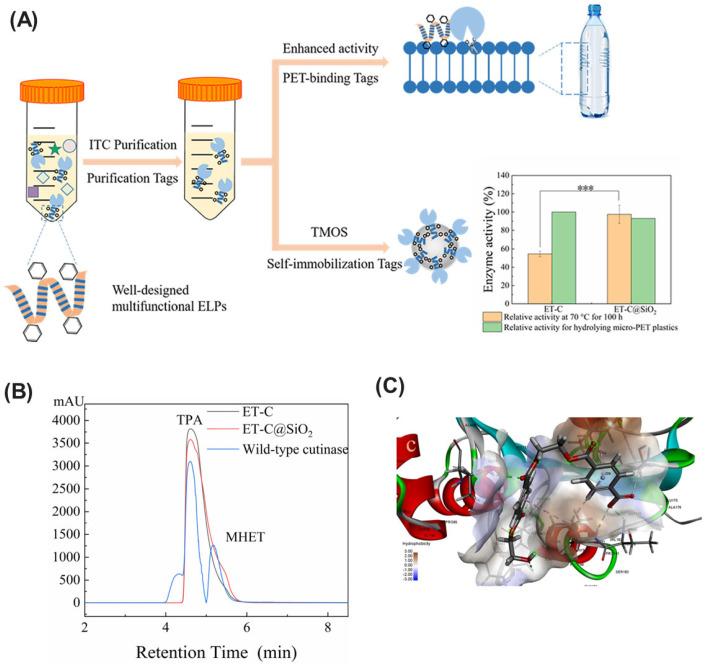
Cutinase immobilization and PET degradation applications. (**A**) Self-immobilization of ELP-tagged cutinase on silica particles enhances enzyme activity and facilitates PET plastic degradation. *** *p* < 0.001. (**B**) HPLC analysis comparing the effectiveness of immobilized cutinase (ET-C@SiO_2_), ELP-tagged cutinase (ET-C), and wild-type cutinase in degrading PET microplastics. (**C**) The interaction between the model substrate (2PET) and the fusion cutinase (ET-C). (**A**–**C**): Reproduced with permission from [213], Copyright 2022 Elsevier B.V.

A recent study presents a significant advancement in enzymatic PET degradation by addressing critical limitations through a versatile “all-in-one” strategy utilizing elastin-like polypeptide (ELP) tags [213]. Conventional methods faced hurdles such as intricate enzyme preparation, restricted access to PET substrates, and poor reusability of free enzymes, further complicated by occasional inhibition from intermediate products. The researchers successfully engineered ELP-tagged cutinase (ET-C), enabling efficient and scalable preparation via centrifugation with high activity recovery and yield (Figure 9). This tagged enzyme exhibited enhanced activity compared to the untagged counterpart, effectively degrading both micro and macro-sized PET plastics. Furthermore, the ELPs facilitated the self-immobilization of the cutinase onto silica (ET-C@SiO_2_), resulting in a robust biocatalyst with superior loading capacity, activity, and thermal stability. Notably, the immobilized enzyme displayed remarkable reusability, retaining significant activity after multiple cycles. Furthermore, the ELP tags directed the degradation process toward terephthalic acid (TPA) production, circumventing the formation of inhibitory intermediates (MHET) observed with the untagged enzyme. Collectively, these findings highlight the potential of ELP tags as a practical and scalable approach to address the challenges associated with enzymatic PET degradation. This strategy offers a promising avenue for developing more economical and efficient bioremediation solutions to combat plastic pollution.

A promising approach to enhancing polyethylene terephthalate (PET) degradation utilizes cross-linked enzyme aggregates (CLEA) [233]. To address the limitations of free PETase, a biocatalyst with significant potential for plastic bioremediation, Lee et al. developed PETase-Amy-CLEA. By incorporating amylopectin (Amy) as a cross-linker, the researchers significantly improved enzyme performance. PETase-Amy-CLEA exhibited superior thermal and pH stability compared to its free counterpart, enabling broader operational conditions. Moreover, the immobilized enzyme demonstrated exceptional reusability, retaining over 70% of its activity after repeated cycles. Notably, PETase-Amy-CLEA achieved a 66.7% higher product yield in PET degradation compared to the free enzyme. Although the immobilized enzyme exhibited slightly lower catalytic efficiency, its enhanced stability and reusability make it a promising candidate for large-scale PET plastic biodegradation. The study underscores the potential of computational modeling in identifying optimal cross-linkers for developing even more effective CLEA-based enzymes.

**Figure 10:**
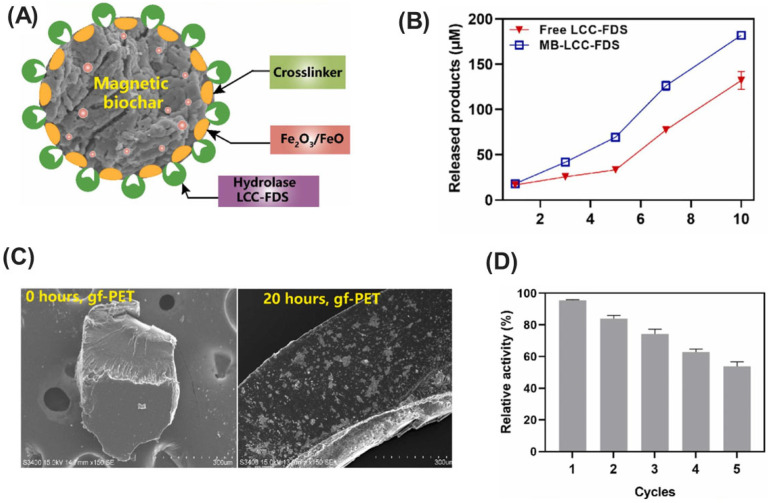
Enhanced PET plastic degradation using magnetic biochar-immobilized hydrolase. (**A**) Fabrication process for PET hydrolase immobilization on magnetic biochar (MB) using glutaraldehyde cross-linking. (**B**) Comparison of the degradation efficiency of free and immobilized enzymes on PET plastic. (**C**) SEM images showing the visual transformation of PET microplastics after 20 h of treatment with MB-LCC-FDS. (**D**) Reusability of the immobilized enzyme after five consecutive degradation cycles. (**A**–**D**): Reproduced with permission from [214], Copyright 2024 Elsevier B.V.

Han et al. present a significant advancement in bioremediating soil microplastic pollution through magnetic biochar-immobilized PET hydrolase (MB-LCC-FDS) [214]. MB-LCC-FDS demonstrated superior performance compared to its free enzyme form, exhibiting enhanced relative activity and reusability (Figure 10). In soil microcosm experiments, MB-LCC-FDS effectively degraded polyethylene terephthalate microplastics (PET-MPs) by 29.6%, converting them into a readily metabolizable intermediate (mono(2-hydroxyethyl) terephthalate, MHET) for native soil microbes. This approach not only targeted PET-MP removal but also fostered a positive response within the soil microbiome. MB-LCC-FDS treatment demonstrably altered the functional composition of soil microbiota, promoting beneficial bacteria like *Microbacteriaceae* and *Skermanella* while reducing others. Notably, the addition of MB-LCC-FDS enhanced crucial soil functions related to nitrogen fixation, phosphorus uptake, and organic matter decomposition while mitigating processes like denitrification and nitrification. This research highlights the synergistic potential of immobilized enzymes and soil microbes for microplastic degradation. Furthermore, it sheds light on the positive influence this approach has on soil health by regulating key nutrient cycles. Overall, the study presents a novel and sustainable solution for bioremediating soil microplastics, promoting a healthier and more functional soil ecosystem.

A recent study developed a promising biocatalyst for BHET degradation, a key product of polyethylene terephthalate (PET) plastic breakdown, targeting microplastic pollution in wastewater treatment [215]. This approach utilized enzyme immobilization within metal–organic frameworks (MOFs), creating CrL_MOFs. These CrL_MOFs incorporated *C. rugosa* lipase enzyme specifically designed to degrade plastic. They tackled BHET through a two-fold strategy: the immobilized enzyme broke down BHET molecules while the MOF structure itself adsorbed the byproducts. This combined approach resulted in superior BHET removal efficiency compared to using free enzymes alone. An additional benefit of CrL_MOFs was their reusability, addressing a major drawback of single-use enzymes and offering a more economical solution. The potential existed for CrL_MOFs to not only target BHET but also co-adsorb other plastic byproducts and pollutants, enabling the removal of multiple contaminants in a single step during wastewater treatment. This research paved the way for more efficient and sustainable strategies to combat plastic pollution in our waterways.

Further study introduces a significant advancement in the enzymatic degradation of nanoplastics in water. Conventional approaches utilizing enzymes like cutinase face limitations. To address this, researchers developed a microreactor system featuring immobilized cutinase on Janus microspheres [234]. These microspheres, self-assembled within microfluidic droplets, offer high enzyme loading capacity and unique dual-porosity properties. Despite slightly lower activity compared to the free enzyme, the immobilized cutinase exhibits comparable degradation performance for nanoplastics and demonstrates good reusability over multiple cycles. This microreactor strategy highlights the potential of immobilized enzymes for more efficient and cost-effective solutions in enzymatic nanoplastic remediation, paving the way for improved water treatment methods.

### 4.5. Degradation of Industrial Chemical Pollutants with Immobilized Enzymes

A wide range of persistent chemical pollutants are generated by industrial activities, posing significant environmental and human health risks. These pollutants, including phenolics utilized in plastic production, phthalate ester plasticizers, and harmful byproducts like BTEX compounds and benzo[a]pyrene, exhibit bioaccumulation tendencies within living organisms. The presence of halogenated solvents, such as 1,2,3-trichloropropane (TCP), further complicates environmental remediation efforts. Fortunately, immobilized enzymes offer a promising and sustainable solution for mitigating these contaminants. By adopting immobilized enzymes, industries can significantly reduce their environmental footprint and contribute to the protection and restoration of natural ecosystems.

**Figure 11:**
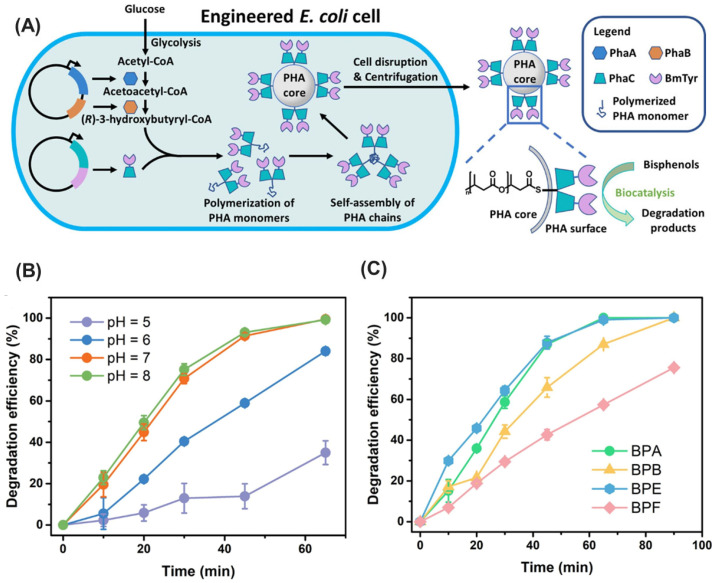
Engineering and performance of PHA-BmTyr for bisphenol removal. (**A**) Schematic depicting PHA-BmTyr biocatalyst creation. Three enzymes, PhaA (β-kethothiolase), PhaB (acetoacetyl-CoA reductase), and PhaC (PHA synthase), self-assemble to form a support matrix for *Bacillus megaterium* tyrosinase. (**B**) Degradation efficiency of PHA-BmTyr for bisphenol A (BPA) at different pH values. (**C**) Real-world applicability of PHA-BmTyr for bisphenol removal from secondary wastewater effluent. (**A**–**C**): Reproduced with permission from [77], Copyright 2022 Elsevier Ltd. (CC BY-NC-ND 4.0).

Bisphenol A (BPA), an endocrine-disrupting chemical with negative health and environmental impacts, is a growing concern in water pollution. In a recent study, a genetically engineered biocatalyst, PHA-BmTyr, was investigated for the remediation of BPA and related contaminants (Figure 11) [77]. The PHA-BmTyr biocatalyst, created by attaching *B. megaterium* tyrosinase (BmTyr) onto self-assembled biopolymer polyhydroxyalkanoate (PHA) beads, offers a one-step production method. This biocatalyst effectively degraded various BPA analogs, significantly reducing their estrogenic activity and generating less toxic byproducts. Moreover, PHA-BmTyr exhibited exceptional reusability and stability, maintaining high activity after multiple cycles and during storage. Notably, it effectively degraded BPA analogs even in real wastewater samples. This research presents a promising approach for sustainable water treatment by utilizing a previously underexplored enzyme (tyrosinase) with a simple production method, high stability, reusability, and real-world applicability for BPA and similar contaminant removal.

Expanding on BPA degradation with immobilized enzymes, Zayed et al. investigated the use of immobilized laccase enzymes as a promising approach for efficient BPA removal [235]. They developed novel supporting materials (NH2-PMMA and NH2-PMMA-Gr) specifically designed for laccase immobilization. The resulting immobilized laccase exhibited significantly improved stability compared to the free enzyme. This enhanced stability encompassed broader optimal pH and temperature ranges for activity, extended storage duration, and remarkable reusability (up to 86.7% activity after 10 cycles). Consequently, the immobilized laccase demonstrated superior BPA degradation efficiency. Even after five reuse cycles, it effectively degraded over 77% of BPA, highlighting the potential of this approach for developing sustainable and efficient methods for BPA removal from wastewater.

**Figure 12:**
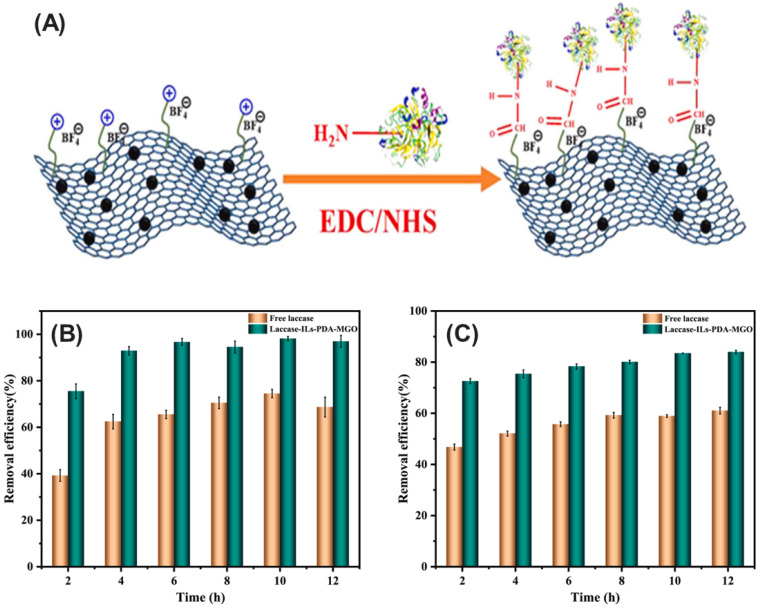
(**A**) Schematic of laccase biocatalyst preparation using ionic liquids, polydopamine, and magnetic graphene oxide (MGO). Removal efficiency of (**B**) 2,4-DCP and (**C**) BPA by free and immobilized laccase. (**A**–**C**): Reproduced with permission from [236], Copyright 2024 Elsevier Ltd.

A significant advancement in bioremediation of phenolic pollutants from wastewater was achieved through the development of a magnetically recoverable, immobilized laccase enzyme. Zhang et al. addressed the well-documented challenge of graphene-induced enzyme inactivation due to electrostatic interactions (Figure 12). To circumvent this limitation, they engineered a novel carrier material composed of magnetic graphene oxide flakes functionalized with ionic liquids (ILs) as spacer arms and coated with polydopamine (PDA) [236]. These IL spacers act as a crucial barrier, effectively preventing detrimental interactions between the laccase enzyme and the underlying graphene surface. The resulting immobilized laccase (laccase-ILs-PDA-MGO) exhibited remarkable stability and reusability, surpassing the performance of the free enzyme. Notably, laccase-ILs-PDA-MGO retained high activity across a broader temperature range (including elevated temperatures of 50 °C) and a wider pH spectrum. Furthermore, this immobilized laccase demonstrated exceptional efficiency in removing phenolic pollutants (2,4-DCP and BPA) from water, achieving over 97% removal within 12 h. Perhaps most significantly, the magnetic properties of the carrier material allowed for facile separation and reuse of the laccase-ILs-PDA-MGO for multiple cycles. Even after seven cycles, the immobilized enzyme maintained a remarkable removal efficiency exceeding 80% for both pollutants. This study underscores the transformative role of laccase immobilization using IL spacer arms. This approach enables the development of highly stable and reusable biocatalysts, providing a promising strategy for efficient and sustainable wastewater treatment, ultimately contributing to cleaner industrial processes.

A recent study describes the development of a novel biocatalyst for the degradation of Benzo[a]pyrene (BaP), a pollutant of significant concern [216]. The researchers achieved this by immobilizing laccase, a powerful enzyme, onto a specifically designed magnetic carrier (Fe_3_O_4_@d-SiO_2_@p-SiO_2_). This innovative design offers several advantages, including high laccase loading capacity, enhanced stability across various parameters (pH, temperature, and storage), facile magnetic separation for post-treatment retrieval, and remarkable reusability (over 58% activity after 10 cycles). The study focused on BaP degradation and demonstrated the remarkable effectiveness of the immobilized laccase. It achieved an outstanding 99% BaP removal within 1 h and maintained significant efficiency (over 35%) even after multiple uses. This strongly suggests that the improved stability and reusability of the immobilized laccase translated to superior biodegradation performance. The study further explored the degradation mechanism, revealing a combination of adsorption and enzymatic degradation by the laccase. Overall, this research demonstrates the potential of immobilized laccase on Fe_3_O_4_@d-SiO_2_@p-SiO_2_ as a highly effective tool for bioremediating BaP-contaminated environments.

Phthalate diesters (PAEs), commonly used as plasticizers, are endocrine-disrupting chemicals that pose a threat to human health and the environment. Balci et al. investigated a green approach for PAE removal using immobilized enzymes [237]. Bionanocomposite beads were developed by immobilizing *B. subtilis* esterase onto halloysite nanotubes and encapsulating them within chitosan or alginate beads. Batch degradation tests identified the chitosan-based bionanocomposite as the most effective, achieving complete DBP degradation and nearly 90% DEHP degradation. Furthermore, continuous flow reactors containing both composites demonstrated superior performance with the chitosan composite, completely removing DBP and achieving over 85% DEHP removal across various flow rates. These findings suggest the potential of these bionanocomposites for efficient phthalate diester removal. The chitosan composite, exhibiting exceptional performance and stability, emerges as a promising biocatalyst for the remediation of phthalate ester-contaminated environments.

**Figure 13:**
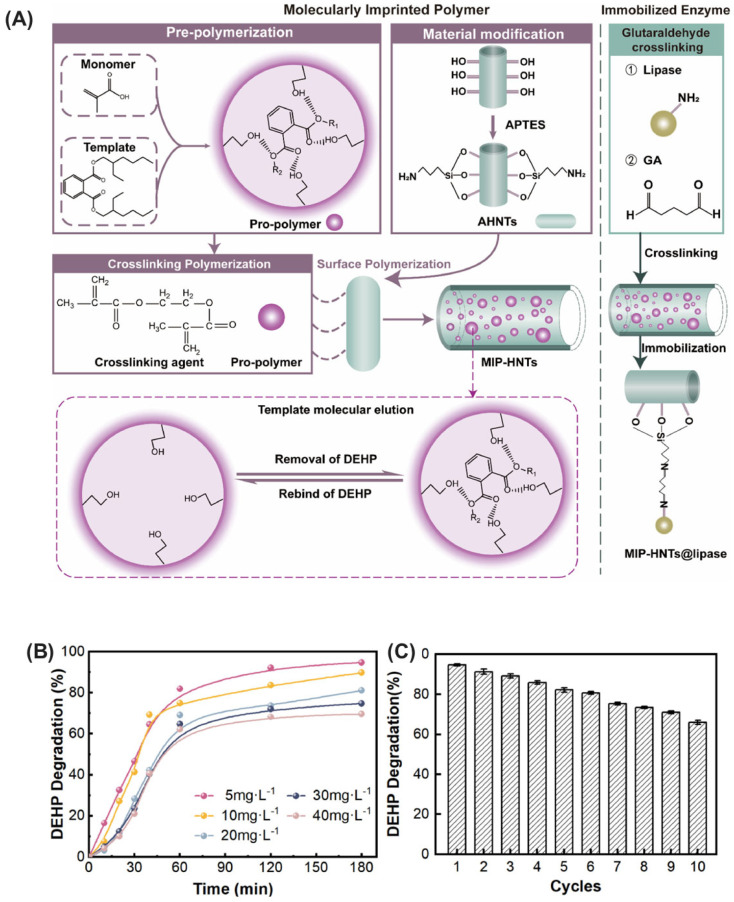
(**A**) Immobilization of *Candida lipolytica* lipase onto MIP-HNTs. (**B**) DEHP degradation efficiency of MIP-HNTs@lipase over varying incubation times (initial DEHP concentration: 5–40 mg·L^–1^). (**C**) Reusability performance of MIP-HNTs@lipase. (**A**–**C**): Reproduced with permission from [217], Copyright 2022 Elsevier B.V.

Another study explored a novel approach for enzymatic degradation of PAEs. This research focused on the immobilization *Candida lipolytica* lipase onto a specially designed carrier (MIP-HNTs) derived from halloysite nanotubes (Figure 13) [217]. The key innovation lies in the use of molecular imprinting, a technique that tailors the MIP-HNTs to recognize DEHP, a prevalent PAE. The resulting immobilized enzyme (MIP-HNTs@lipase) demonstrated high loading efficiency (120.540 mg/g) and enhanced affinity for the target pollutant. This biocatalyst displayed optimal activity under controlled conditions (pH 9 and 50 °C) and demonstrated both good stability and efficient DEHP degradation. Notably, at a DEHP concentration of 5 mg/L, it achieved an impressive degradation rate exceeding 94.7%. Furthermore, the immobilized enzyme maintained significant activity (over 65% degradation rate) even after multiple reuse cycles (10 cycles), highlighting its reusability. This study underscores the potential of combining molecular imprinting and immobilized enzyme technology for PAE degradation. The developed biocatalyst effectively addresses the limitations of free enzymes, namely instability and recycling challenges, while achieving efficient DEHP identification and degradation. This research opens new avenues for the development of biocatalysts with broader applications in the field of pollutant degradation.

A recent study investigated a promising strategy for degrading organic pollutants by employing a biocatalytic system immobilized on nanoparticles. The research focused on the P450 BM3 enzyme, produced by genetically engineered *E. coli*, immobilized onto hollow nanospheres composed of titanium dioxide and copper (TiO_2_-Cu) [218]. Successful immobilization was verified through scanning electron microscopy and enzyme activity assessment. Notably, the immobilized enzyme exhibited a two-fold increase in activity compared to its free counterpart. Furthermore, the P450 BM3-hollow nanosphere biocatalyst effectively degraded isopropanol, a model organic pollutant, under visible light irradiation. This system achieved a remarkable degradation rate (95%) at ambient temperature and neutral pH, significantly outperforming both the free enzyme and the bare hollow nanospheres. These findings suggest that the TiO_2_-Cu composite not only enhanced the immobilized enzyme’s stability and activity but also offered distinct advantages. First, it addressed a major limitation of free enzymes by improving their stability. Second, the photocatalytic properties of the hollow nanospheres eliminated the requirement for expensive cofactors, making the system more cost-effective. Overall, this study demonstrates the potential of TiO_2_-Cu hollow nanospheres as a robust platform for large-scale immobilization of P450 BM3, resulting in enhanced enzyme properties and reusability. This novel photo-nanobiocatalyst holds significant promise for addressing industrial air pollution challenges.

**Figure 14:**
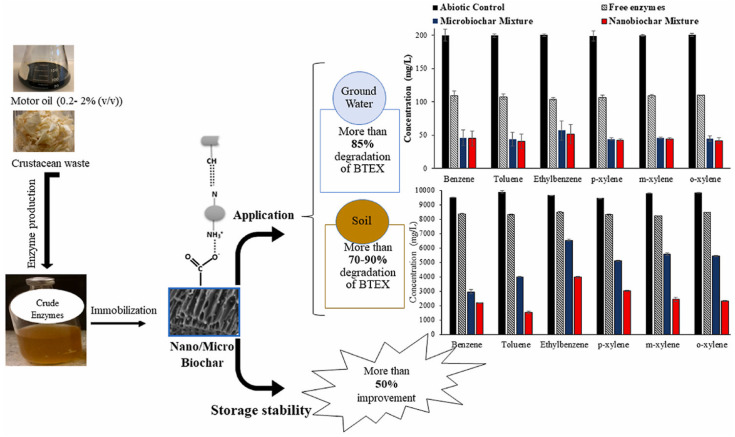
Enhanced BTEX degradation via immobilized enzymes on biochar matrices. The production and subsequent immobilization of two enzymes, toluene/o-xylene monooxygenase (ToMO) and catechol 1,2-dioxygenase (C1,2D), specifically designed for BTEX degradation. (BTEX = benzene, toluene, ethylbenzene, and xylenes). The effectiveness of free and immobilized enzymes on micro and nano biochar-chitosan matrices for BTEX degradation is compared. Control (1) represents BTEX contamination without enzymes or matrices (baseline). Control (2) represents BTEX contamination with deactivated immobilized enzymes. Reproduced with permission from [219], Copyright 2021 Elsevier Ltd.

Aromatic pollutants, including BTEX (benzene, toluene, ethylbenzene, and xylenes), are persistent environmental contaminants with detrimental effects on human health. Miri et al. investigated a novel approach for BTEX biodegradation employing immobilized cold-active enzymes. This study utilized toluene/o-xylene monooxygenase (ToMO) and catechol 1,2-dioxygenase (C1,2D) enzymes produced by a newly isolated psychrophilic bacterium, *Pseudomonas* S2TR-14 (Figure 14) [219]. These enzymes were successfully immobilized within micro/nano biochar-chitosan matrices. Interestingly, the presence of used motor oil in the growth medium demonstrably enhanced enzyme production. The immobilization process achieved a high yield exceeding 74% and significantly improved the enzymes’ storage stability, with over 50% residual activity remaining after 30 days. Furthermore, the immobilized enzymes effectively degraded BTEX in both groundwater and soil samples, achieving over 80% removal efficiency at low temperatures (10 °C). This study underscores the potential of co-immobilizing cold-active enzymes onto biochar-chitosan matrices for efficient BTEX biodegradation in cold environments. This approach offers a promising strategy for the remediation of contaminated groundwater and soil.

A novel approach was developed to address the degradation of halogenated pollutants, a class of hazardous environmental contaminants. The research focused on enhancing the application of halohydrin dehalogenase (HHDH), a promising enzyme for biodegradation [220]. By immobilizing HHDH on functionalized magnetic biochar, the scientists created a new biocatalyst named HheC-N-MBC_600_. This immobilization process yielded several advantages compared to the free enzyme. The HheC-N-MBC_600_ biocatalyst retained a high level of activity (85%) and exhibited exceptional storage stability, retaining 50% activity after 70 days at 4 °C (compared to a mere 8% for the free enzyme). Furthermore, the immobilized enzyme demonstrated remarkable tolerance to organic solvents and reusability, maintaining over 70% activity after 30 consecutive reuse cycles. Importantly, HheC-N-MBC_600_ preserved the same enantioselective behavior as the free enzyme. When combined with an immobilized epoxide hydrolyase enzyme (EchA-MBC_600_), HheC-N-MBC_600_ successfully converted a toxic compound (1,3-dichloro-2-propanol) into a non-toxic product. Overall, this research highlights the significant impact of immobilization on HHDH. This approach transforms HHDH into a robust, reusable biocatalyst with promising applications in both environmental pollutant degradation and the production of valuable chiral compounds. The magnetic biochar carrier offers additional benefits of easy separation and environmentally friendly characteristics. These findings suggest that HheC-N-MBC_600_ has great potential for industrial biocatalysis.

**Figure 15:**
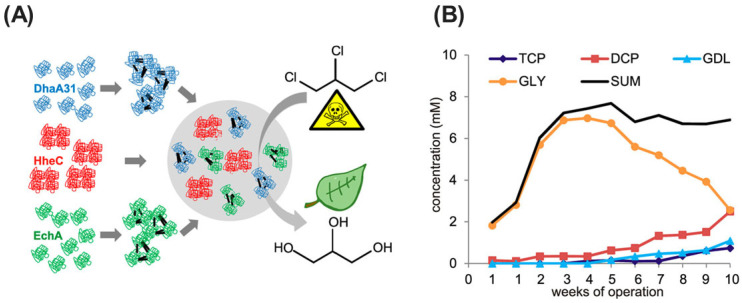
Continuous biodegradation of 1,2,3-Trichloropropane (TCP). (**A**) Immobilized enzymes derived from specific bacteria within PVA particles enable continuous operation in a packed-bed reactor. The enzymatic cascade involves haloalkane dehalogenase, haloalcohol dehalogenase, and epoxide hydrolase for efficient TCP degradation. (**B**) Tracking the remaining amounts of intermediate products (2,3-dichloropropane-1-ol, DCP; glycidol, GDL) and the final product (glycerol, GLY) formed during TCP degradation over 10 weeks, measured in the effluent vessels of the packed-bed reactor. (**A**,**B**): Reproduced with permission from [238], Copyright 2014 American Chemical Society.

A novel bioremediation approach was investigated for the degradation of 1,2,3-trichloropropane (TCP), a toxic industrial contaminant found in groundwater [238]. Dvorak et al. employed an immobilized synthetic pathway comprised of three strategically engineered enzymes: haloalkane dehalogenase from *Rhodococcus rhodochrous* and haloalcohol dehalogenase and epoxide hydrolase, both derived from *Agrobacterium radiobacter* AD1 (Figure 15). This enzymatic cascade effectively converts TCP into harmless glycerol. Notably, the enzymes were encapsulated within PVA particles for continuous operation in a packed-bed reactor. This immobilization strategy offered significant advantages. Firstly, it enabled the treatment of concentrated TCP solutions (up to 10 mM), exceeding the tolerance limit of living microorganisms. Secondly, the immobilized enzymes demonstrated versatility by functioning effectively in both batch and continuous systems. A packed bed reactor containing the immobilized biocatalysts achieved continuous TCP conversion to glycerol over a 2.5-month period, maintaining high efficiency (97% conversion to intermediates and 78% conversion to final product). This research highlights the potential of immobilized enzymes for the efficient decontamination of TCP-contaminated groundwater, offering a promising strategy for environmental remediation efforts.

The development of highly sensitive and selective biosensors for environmental monitoring is an ongoing pursuit, particularly for the detection of hazardous pollutants like halogenated organic compounds in water samples. Shahar et al. presented a significant contribution to this field by introducing a novel reflectometric biosensor that leverages immobilized enzymes for the indirect quantification of these contaminants [239]. Haloalkane dehalogenase (DhlA) enzyme was covalently attached to strategically modified polyacrylate microspheres, while a chromoionophore dye (NBC) was physically immobilized on the same platform to function as a colorimetric indicator. The enzymatic breakdown of the pollutant by the immobilized DhlA triggered a color change in the NBC dye, enabling detection via reflectance spectrophotometry. This research underscores the transformative role of immobilized enzymes in creating a novel and effective biosensor with remarkable performance characteristics. The rapid response times, exceptional stability, and reliable detection capabilities exhibited by this biosensor position it as a promising tool for water and wastewater surveillance within industrial sectors.

## 5. Conclusions and Future Outlooks

The growing threat of micropollutant contamination in our environment necessitates the development of sustainable and effective remediation strategies. These contaminants, present at low concentrations yet persistent in nature, pose significant risks to environmental and human health. Conventional remediation methods often fall short due to limitations in effectiveness or environmental sustainability. This review highlights the immense potential of microbial enzymes as a promising alternative for environmental cleanup. Their diverse substrate specificity, biodegradability, and remarkable ability to degrade a wide range of pollutants position them as a powerful tool for bioremediation. However, limitations in operational stability and reusability can hinder their practical application.

Immobilization techniques emerge as a transformative strategy to overcome the limitations of free enzymes for bioremediation. By anchoring enzymes onto inert supports, immobilization enhances their stability and reusability. This translates to improved efficiency in pollutant degradation and cost-effective bioremediation processes. This targeted approach allows for the development of effective strategies to address a wide range of environmental challenges. These include the degradation of pharmaceuticals, dyes, pesticides, microplastics, and industrial chemical pollutants such as phenolics, phthalate esters, and BTEX compounds. This review has showcased the effectiveness of immobilized enzymes in degrading a diverse range of environmental pollutants. From persistent pharmaceutical residues to microplastics and complex industrial chemicals, immobilized enzymes offer a promising and sustainable solution for environmental restoration.

The future of enzymatic bioremediation for environmental cleanup brims with exciting possibilities. Researchers are actively pursuing novel immobilization techniques that can significantly enhance enzyme efficiency, reusability, and long-term stability. For instance, exploring novel materials with tailored properties for enzyme attachment holds promise for even more outstanding biocatalytic performance. These advancements are crucial for ensuring the long-term viability and cost-effectiveness of enzyme-based bioremediation solutions, ultimately promoting their adoption as a sustainable approach to environmental restoration.

The field of enzyme engineering holds immense potential to broaden the capabilities of enzymatic bioremediation. By engineering enzymes to target emerging pollutants, particularly complex industrial chemicals, researchers can expand the range of contaminants susceptible to biodegradation. Envision the development of enzymes specifically engineered to degrade newly identified pollutants, facilitating a proactive approach to environmental remediation. Furthermore, investigations into the integration of microbial enzymes with complementary technologies, such as membrane filtration or advanced oxidation processes, offer the potential for developing comprehensive environmental cleanup solutions specifically tailored for industrial pollutants. Combining enzymatic biodegradation with these complementary technologies could lead to the development of more efficient and targeted remediation strategies, allowing for a more holistic approach to tackling environmental challenges.

To achieve broader adoption of enzyme-based bioremediation for large-scale industrial wastewater treatment, it is essential to address its economic feasibility and scalability. Research should focus on optimizing production processes and enhancing cost-effectiveness to make this technology more viable for industrial applications. Life cycle assessment (LCA) studies are crucial in evaluating the environmental and economic footprint of enzyme-based bioremediation compared to traditional methods, providing comprehensive insights into its benefits and drawbacks. Additionally, a thorough evaluation of the broader environmental impact is necessary, considering potential unintended consequences such as the introduction of novel enzymes into ecosystems. By carefully assessing these risks, researchers can ensure the sustainable implementation of enzyme-based bioremediation.

Finally, machine learning (ML) presents a transformative frontier for bioremediation. By integrating vast datasets on pollutant characteristics, enzyme properties, and environmental conditions, ML algorithms can accelerate the development of optimized bioremediation protocols. As ML capabilities continue to advance, its role in streamlining and optimizing bioremediation strategies will become increasingly significant. ML algorithms can predict ideal enzyme-pollutant combinations, determine suitable immobilization methods for specific applications, and even fine-tune environmental conditions to maximize biodegradation rates. This data-driven approach has the potential to revolutionize bioremediation, paving the way for a more sustainable future.

By outlining the current state of research and future outlooks, this review underscores the exciting potential of immobilized enzymes for environmental restoration. Through continuous research and development in novel immobilization techniques, enzyme engineering, integration with complementary technologies, and the application of machine learning, enzymatic bioremediation holds immense promise for tackling the multifaceted challenge of micropollutant contamination. This sustainable approach offers a powerful tool for environmental restoration, promoting a healthier environment for all.

## Figures and Tables

**Table 1:** Emerging applications of immobilized microbial enzymes for mitigating multiple pollutants.

Enzyme	Microbial Source	Support Matrix	Immobilization Method	Advantages of Immobilization	Mitigation Application	References
EreB esterase	*E. coli*, recombinantly produced	Enzymatic membrane reactor (EMR)	Adsorption	Long-term stability, continuous degradation	Continuous erythromycin degradation at a rate of 15.8 mg/h for 100 h	[195]
EreB esterase	*E. coli*, recombinantly produced	Palygorskite (acid-modified)	Cross-linking	Enhanced stability (temperature and pH), increased activity, reusability (45% after 10 cycles)	Erythromycin degradation in polluted water (20 mg/L)—achieved degradation within 300 min	[196]
EreB esterase	*E. coli*, recombinantly produced	Cu-BTC MOF	Adsorption	Improved heat tolerance (25–45 °C), improved pH tolerance (6.5–10), increased substrate affinity, reusability (57% activity after 10 cycles)	Complete inactivation of erythromycin in wastewater	[197]
Laccase	*Trametes versicolor*	HMCs	Covalent interaction and physical adsorption	Improved stability (temperature, pH, storage), reusability, high removal efficiency for antibiotics (TCH and CPH)	Bioremediation of antibiotic pollutants in environmental application	[198]
Laccase	*Bacillus amyloliquefaciens,* recombinantly produced in *E. coli*	Inorganic hNFs	Sonication-mediated self-encapsulation	Enhanced thermal and storage stability, superior degradation performance for various TCs (including tigecycline), reusability, reduced TC toxicity	Removal of tetracycline antibiotics (TCs) from the environment	[199]
Laccase	*Cerrena unicolor*	Magnetic CLEAs	Cross-linking	Potentially improved stability and reusability	Complete degradation of tetracycline (TC) and oxytetracycline (OTC) (100 μg/mL) within 48 h (pH 6, 25 °C), reduced antimicrobial activity of TC and OTC	[200]
Laccase	*Trametes versicolor*	Magnetic biochar	Adsorption and cross-linking method	Improved pH, thermal, storage, and operational stability	Synergistic effect of adsorption by MBC and degradation by laccase for higher removal of quinolone antibiotics from wastewater	[201]
Laccase	*Trametes versicolor*	3D printed PLA scaffolds	Physical adsorption	Improved chemical and thermal stability, reusability	Removal of estrogens (estradiol and ethinylestradiol) from real wastewater	[202]
Laccase, versatile peroxidase, and glucose oxidase	*Trametes versicolor* (for laccase); *Bjerkandera adusta* (for peroxidase); *Aspergillus niger* (for glucose oxidase)	-	Cross-linking (Combi-CLEAs)	Retained enzyme activity (30–40% each), broader degradation range	Treatment of municipal wastewater effluents	[203]
Manganese peroxidase	*Aspergillus flavus*	Iron oxide nanoparticles	Physical adsorption	Enhanced thermal stability, wider pH and temperature range, improved catalytic activity, magnetic separation and reusability	Textile wastewater treatment, Direct Red 31 (complete decolorization), Acid Black 234 (92% decolorization)	[204]
Manganese peroxidase	*Phanerochaete chrysosporium*	Silica gels	Encapsulation through the sol-gel method	4-fold increase in enzymatic activity via co-immobilization with Mn^3+^	Dye decolorization (the co-immobilized system degraded 81.1% of RB19 and 32.7% of AO7)	[205]
Laccase	*Bacillus subtilis*	Calcium phosphate	Co-encapsulation of laccase and ABTS via biomineralization	High activity recovery, enhanced pH tolerance, improved storage stability	MG dye degradation in wastewater	[206]
Tyrosinase	*Agaricus bisporus*	3-mercaptopropionic acid modified silver-coated Fe_3_O_4_ nanoparticles	EDC/NHS chemistry (covalent)	Increased substrate affinity (1.4x), improved storage stability (68.3% after 84 days), reusability (48.9% after 6 cycles)	Azo dyes (Reactive Green 19, Congo Red), phenolic compounds (phenol, bisphenol F, bisphenol A, p-cresol, phenyl acetate, chlorophenols)	[207]
EstM160K (engineered esterase)	*Geobacillus uzenensis*	Epoxy resin lx-105s	Covalent bonding	Enhanced thermostability (T½ 36.8 h at 70 °C), reusability	Malathion pesticide: 95.8% at 20 mg/L, Bifenthrin pesticide: 90.4% at 500 mg/L (packed-bed column reactor)	[208]
PTE	*Sulfolobus solfataricus*	Specialized biocatalytic membrane	Covalent immobilization	Maintain activity through cycles with surfactant reloading, 96% paraoxon conversion rate in biocatalytic reactor	Degradation of paraoxon (organophosphate pesticide)	[209]
Laccase	*Coriollopsis gallica*	MSU-F	Physical adsorption	Potentially reduces genotoxicity and apoptotic effects, reduces binding to hormone receptors	Degradation of dichlorophen pesticide	[210]
Laccase, Aryl alcohol oxidase, Lignin peroxidase, Manganese peroxidase	*Pleurotus ostreatus*	Nano-silica particles	Covalent immobilization	Enhanced stability, reusability, wide pH and temperature range (4–9, 20–55 °C)	Complete elimination of p,p′-DDT within 12 h (pH 5, 30 °C)	[211]
PETase	*Ideonella sakaiensis*	Co_3_(PO_4_)_2_ nanostructures	Encapsulation through biomimetic mineralization	Increased enzyme loading, improved stability (broader temperature and pH tolerance), reusability	Bioremediation of PET plastic pollution (by degrading to terephthalic acid)	[212]
ELP-tagged cutinase	Synthetic construct, recombinantly produced in *E. coli*	Biomimetic silica	Self-immobilization via ELPs	Superior loading capacity, activity, and thermal stability	Bioremediation of PET plastic pollution	[213]
PET hydrolase	Recombinant *E. coli* expressed LCC-FDS	Magnetic biochar	Covalent immobilization	Enhanced relative activity, improved reusability	Bioremediation of PET microplastics in soil	[214]
Lipase	*Candida rugosa*	MOFs	Physical adsorption	Enhanced BHET removal efficiency, reusability	Bioremediation of BHET from microplastic pollution in wastewater treatment	[215]
Tyrosinase	*Bacillus megaterium*	Self-assembled biopolymer beads	Genetic immobilization	Degrades various BPA analogues, reduces estrogenic activity, exceptional reusability and stability	Sustainable water treatment for BPA and similar contaminants	[77]
Laccase	*Trametes versicolor*	Multilayer core–shell magnetic mesoporous silica	Physical adsorption	High loading capacity (567 mg/g), improved pH, thermal, and storage stability, easy magnetic recovery and good reusability (58.2% activity after 10 cycles)	Bioremediation of BaP-contaminated sites (high BaP removal efficiency (99.0% within 1 h))	[216]
Lipase	*Candida lipolytica*	Molecularly imprinted halloysite nanotubes (MIP-HNTs)	Cross-linking	Improved recognition of PAEs, high lipase loading efficiency (76%), good stability and reusability	Degradation of PAE pollutants (specifically DEHP)	[217]
Cytochrome P450 BM3 monooxygenase	*Bacillus megaterium,* recombinantly produced in *E. coli*	Hollow TiO_2_-Cu nanospheres (<50 nm)	Physical adsorption	Increased enzyme activity (doubled compared to free enzyme), enhanced stability, high degradation efficiency (95% of isopropanol)	Air pollution remediation (mitigation of isopropanol pollutants)	[218]
ToMO and C1,2D enzymes	*Pseudomonas* S2TR-14	Micro/nano biochar-chitosan	Physical adsorption and covalent bonding	Enhanced storage stability (>50% activity after 30 days), effective degradation of BTEX in groundwater and soil (over 80% removal at 10 °C)	Biodegradation of BTEX pollutants in cold environments	[219]
Halohydrin Dehalogenase	*Agrobacterium radiobacter* AD1, recombinantly produced in *E. coli*	Functionalized magnetic biochar	Covalent immobilization	Exceptional storage stability (50% activity after 70 days at 4 °C), organic solvent tolerance, excellent reusability (over 70% activity after 30 cycles), easy separation (magnetic)	Biodegradation of halogenated pollutants	[220]

Abbreviations: HMCs, hollow mesoporous carbon spheres; TCH, tetracycline hydrochloride; CPH, chloramphenicol hydrochloride; CLEAs, cross-linked enzyme aggregates; hNFs, hybrid nanoflowers; RB 19, reactive blue 19; AO7, acid orange 7; ABTS, 2,2′-azino bis (3-ethylbenzothiazoline)-6-sulfonic acid; MG, malachite green; EDC, 1-Ethyl-3-(3-dimethylaminopropyl)carbodiimide; NHS, N-Hydroxysuccinimide, PTE, phosphotriesterase; Co_3_(PO_4_)_2_, cobalt phosphate; PET, polyethylene terephthalate; MSU-F, mesoporous synthetic silica foam; LCC-FDS, leaf-branch compost cutinase mutant; MOFs, metal–organic framework; BHET, Bis-(hydroxyethyl)terephthalate; BPA, bisphenol A; PAE, phthalic acid esters; DEHP, di(2-ethylhexyl) phthalate; BaP, Benzo[a]pyrene; ToMO, toluene/o-xylene monooxygenase; C1,2D, catechol 1,2-dioxygenase; BTEX, a complex of benzene, toluene, ethylbenzene, and xylenes.
